# Oncofetal reprogramming in tumor development and progression: novel insights into cancer therapy

**DOI:** 10.1002/mco2.427

**Published:** 2023-12-02

**Authors:** Jiangjun Cao, Zhe Zhang, Li Zhou, Maochao Luo, Lei Li, Bowen Li, Edouard C. Nice, Weifeng He, Shaojiang Zheng, Canhua Huang

**Affiliations:** ^1^ West China School of Basic Medical Sciences and Forensic Medicine, and Department of Biotherapy Cancer Center and State Key Laboratory of Biotherapy, West China Hospital Sichuan University Chengdu China; ^2^ Zhejiang Provincial Key Laboratory of Pancreatic Disease the First Affiliated Hospital School of Medicine Zhejiang University Zhejiang China; ^3^ Key Laboratory of Molecular Biology for Infectious Diseases (Ministry of Education) Department of Infectious Diseases the Second Affiliated Hospital Institute for Viral Hepatitis, Chongqing Medical University Chongqing China; ^4^ Department of anorectal surgery Hospital of Chengdu University of Traditional Chinese Medicine and Chengdu University of Traditional Chinese Medicine Chengdu China; ^5^ Department of Biochemistry and Molecular Biology Monash University Clayton VIC Australia; ^6^ State Key Laboratory of Trauma Burn and Combined Injury Institute of Burn Research, Southwest Hospital, Third Military Medical University (Army Medical University) Chongqing China; ^7^ Hainan Cancer Medical Center of The First Affiliated Hospital, the Hainan Branch of National Clinical Research Center for Cancer, Hainan Engineering Research Center for Biological Sample Resources of Major Diseases Hainan Medical University Haikou China; ^8^ Key Laboratory of Tropical Cardiovascular Diseases Research of Hainan Province, Hainan Women and Children's Medical Center, Key Laboratory of Emergency and Trauma of Ministry of Education Hainan Medical University Haikou China

**Keywords:** cancer therapy, drug resistance, embryonic development, tumor development and progression

## Abstract

Emerging evidence indicates that cancer cells can mimic characteristics of embryonic development, promoting their development and progression. Cancer cells share features with embryonic development, characterized by robust proliferation and differentiation regulated by signaling pathways such as Wnt, Notch, hedgehog, and Hippo signaling. In certain phase, these cells also mimic embryonic diapause and fertilized egg implantation to evade treatments or immune elimination and promote metastasis. Additionally, the upregulation of ATP‐binding cassette (ABC) transporters, including multidrug resistance protein 1 (MDR1), multidrug resistance‐associated protein 1 (MRP1), and breast cancer‐resistant protein (BCRP), in drug‐resistant cancer cells, analogous to their role in placental development, may facilitate chemotherapy efflux, further resulting in treatment resistance. In this review, we concentrate on the underlying mechanisms that contribute to tumor development and progression from the perspective of embryonic development, encompassing the dysregulation of developmental signaling pathways, the emergence of dormant cancer cells, immune microenvironment remodeling, and the hyperactivation of ABC transporters. Furthermore, we synthesize and emphasize the connections between cancer hallmarks and embryonic development, offering novel insights for the development of innovative cancer treatment strategies.

## Graphical abstract

Various regulatory mechanisms are involved in the development of a fertilized egg into an embryo to ensure that it proceeds normally. Meanwhile, cancer cells also exhibit several embryo‐like characteristics that enhance multidrug resistance. (A) The level of corpus‐secreted progesterone can be mediated by the inner or outer environment and lead to embryonic diapause. (a) Cancer cells are able to transition to a dormant state after treatment. (B) Wnt, Notch, Hedgehog, and Hippo signaling pathways contribute to development by driving the proliferation and differentiation of embryonic cells. (b) Cancer stem cells are considered drug‐resistant cells associated with some embryonic developmental signaling pathways. (C) Preimplantation embryo was reported to drive the formation of immunosuppressive microenvironment to evade attack from maternal immune system. (c) The immune microenvironment components of cancer cells can confer resistance to immune checkpoint blockades (ICBs). (D) To export environmental toxins and provide a favorable condition for development, ABC transporters are usually overexpressed in the blood–embryo barrier. (d) ABC transporter overexpression in cancer cells results in the development of multidrug resistance.

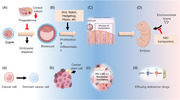

## 1 BACKGROUND

Embryogenesis is a sophisticated process accompanied by rapid cell proliferation, differentiation, and material exchange in early or later development.^1^ During this period, embryonic development‐related pathways, such as Wnt, Notch, hedgehog, Hippo, and transforming growth factor (TGF)‐β signaling, are continuously activated and coordinated with each other to meet the needs of embryonic development.^2^, ^3^ Specifically, the Wnt family regulates embryo polarity and patterning and the morphogenesis of several organs.^4^ Endothelial‐to‐hematopoietic and epithelial‐to‐mesenchymal transitions (EMTs) can both be regulated by Notch signaling. Hedgehog signaling provides positional information and fate instruction to cells.^5^ The Hippo signaling is critical for angiogenesis and vascular development.^6^ In addition, transforming Growth Factor‐β (TGF‐β) signaling cascade contributes to organogenesis.^7^

To cope with harsh conditions for embryonic development, hundreds of mammalian species utilize diapause, a period of suspended development, to avoid the adverse effects of the environment.^8^, ^9^ Moreover, fetuses can help to reprogram immune microenvironment to prevent immunological rejection during implantation.^10^ In addition, to supply a large amount of energy and nutrients required during embryonic development, the exchange of substances between mammalian embryos and mothers must operate rapidly, simultaneously leading to the accumulation of environmental toxins.^11^, ^12^ But in fact, ABC transporters, a class of transmembrane proteins in placental barrier functions and significant reproductive processes, are overexpressed in the placenta and control the efflux of toxic substances.^13^ Although the underlying mechanisms controlling embryonic features need further investigation, these characteristics provide an essential foundation for embryonic development.

**FIGURE 1:**
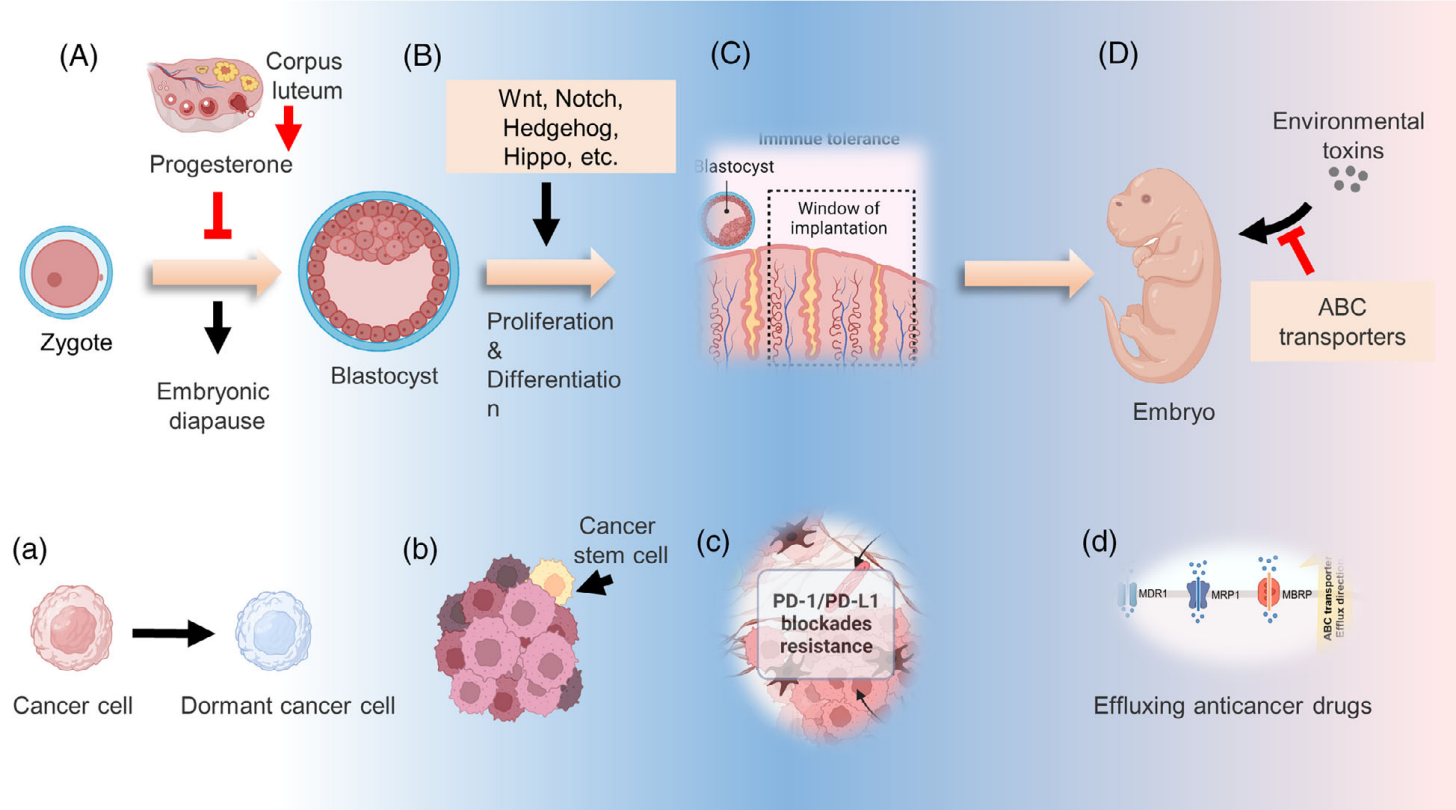
The similar characteristics between embryonic development and cancer development and progression. Various regulatory mechanisms are involved in the development of a fertilized egg into an embryo to ensure that it proceeds normally. Meanwhile, cancer cells also exhibit several embryo‐like characteristics that enhance multidrug resistance. (A) The level of corpus‐secreted progesterone can be mediated by the inner or outer environment and lead to embryonic diapause. (a) Cancer cells are able to transition to a dormant state after treatment. (B) Wnt, Notch, Hedgehog, and Hippo signaling pathways contribute to development by driving the proliferation and differentiation of embryonic cells. (b) Cancer stem cells are considered drug‐resistant cells associated with some embryonic developmental signaling pathways. (C) Preimplantation embryo was reported to drive the formation of immunosuppressive microenvironment to evade attack from maternal immune system. (c) The immune microenvironment components of cancer cells can confer resistance to immune checkpoint blockades (ICBs) such as programmed death‐ligand 1 (PD‐L1)/programmed cell death‐1 (PD‐1). (D) To export environmental toxins and provide a favorable condition for development, ABC transporters are usually overexpressed in the blood–embryo barrier. (d) ATP‐binding cassette (ABC) transporters overexpression in cancer cells results in the development of multidrug resistance.

A growing body of evidence suggests that cancer cells can mimic embryonic traits in development and progression called oncofetal reprogramming^14^, ^15^, ^16^ (Figure 1). First, the dysregulation of embryonic development signaling cascades was frequently detected in cancer cells, which contributes to tumor development and progression.^17^, ^18^, ^19^, ^20^ Targeted therapies focused on developmental pathways have been developed, and many of them have become clinical treatment drugs for various types of cancers.^21^ Meanwhile, the overexpression of ABC transporters plays a role in regulating the tumor immune microenvironment through the transport of various cytokines, thereby influencing antitumor immunity and the sensitivity to anticancer drugs.^22^ The administration of ABC transporter inhibitors in combination with standard chemotherapeutics or immunotherapy could attenuate, at least in part, cancer resistance.^23^, ^24^ Moreover, clinical studies have demonstrated that a small fraction of tumor cells can survive after systemic treatment, exhibiting several characteristics akin to circulating tumor cells, although the underlying mechanism remains a mystery.^25^ Several excellent studies have revealed that these residual tumor cells could transition into an embryonic diapause‐like state, referred to as drug‐tolerant persister cells, cancer stem cells (CSCs), or dormant cancer cells, which contribute to cancer relapse after a period of dormancy.^26^, ^27^, ^28^ While there are no well‐established methods for eliminating these residual cancer cells, understanding how cells enter a quiescent state may provide new therapeutic approaches to cancer recurrence. In addition, the reprogramming of the immune microenvironment endows cancer cells with the ability to escape immune surveillance.^29^

To date, there has been far less emphasis on the systematic revisit of the specific traits that govern tumor development and progression. In this review, we examine in detail how cancer cells mimic developmental‐related features, including the dysregulation of development pathways, embryonic diapause‐like transition, reprogramming of the immune microenvironment, and overexpression of the ABC transporter. Furthermore, we also discuss potential strategies to reverse the tumor embryo‐like state in cancer therapy.

## 2 DYSREGULATION OF EMBRYONIC DEVELOPMENT SIGNALING PATHWAYS IN CANCER

Wnt, Notch, Hedgehog, Hippo, TGF‐β, and Fibroblast growth factor/fibroblast growth factor receptor (FGF/FGFR) signaling are essential for embryonic development. However, abnormal regulation of these pathways usually correlates with the development and progression of numerous cancers.^30^, ^31^, ^32^, ^33^ Here, we review how cancer cells hijack these development‐related pathways.

### 2.1 Reactivation of Wnt signaling pathway

**FIGURE 2:**
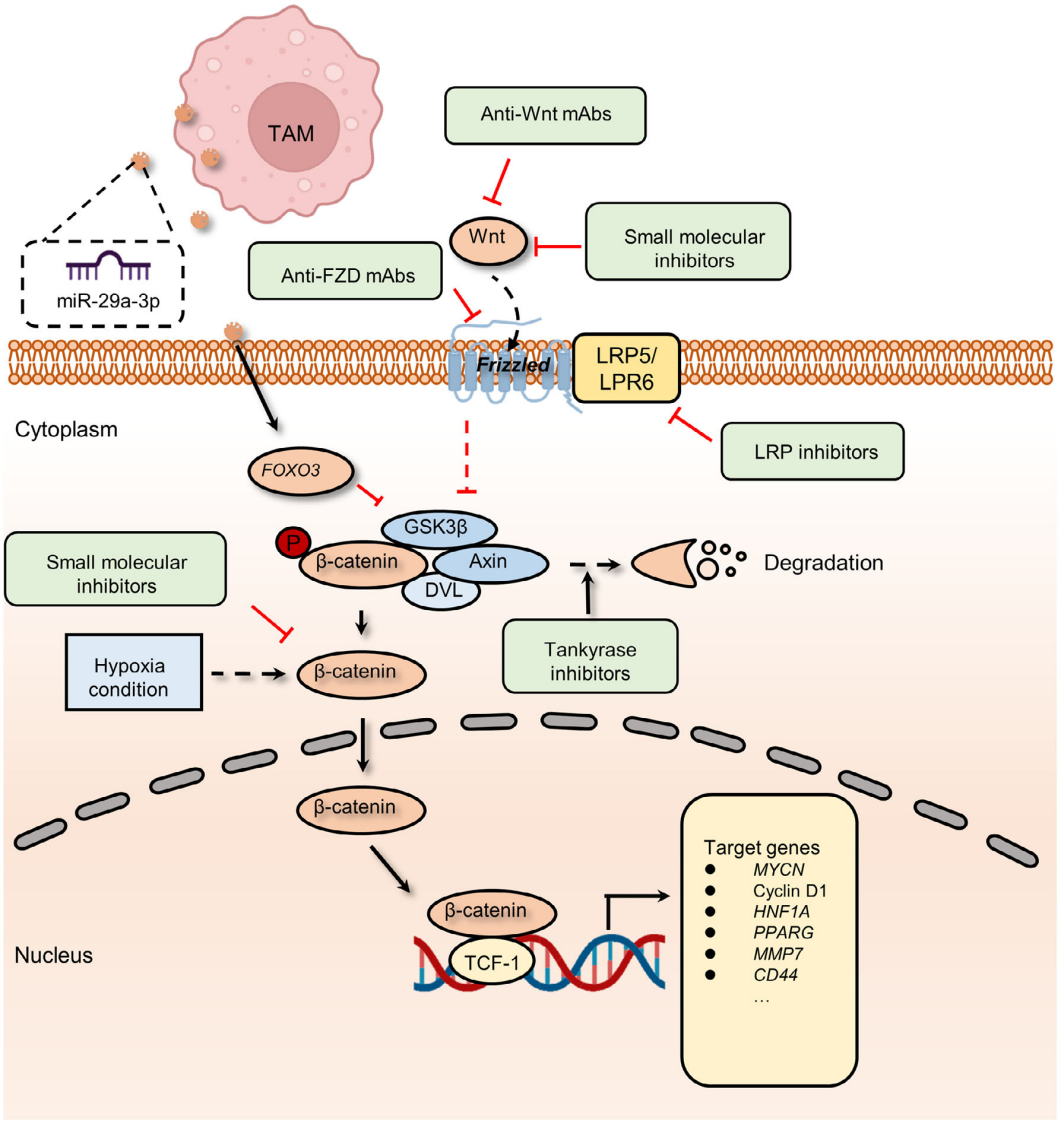
The mechanism of canonical Wnt signaling and related pharmacological inhibitors. The binding of Wnt proteins to Frizzled (Fzd) family receptors can inhibit the phosphorylation of β‐catenin mediated by the destruction complex (mainly including glycogen synthase kinase 3β (GSK3β), Axin, and Dishevelled protein (DVL)) and thereby avoiding degradation. Stable β‐catenin will be translocated into the nucleus and trigger target gene transcription by interacting with TCF‐1 and other factors. Wnt target gene expression, such as *MYCN*, endows resistance to cancer drugs on tumor cells. However, GSK3β could be inhibited by forkhead box O3 (FOXO3), which is activated by tumor‐associated macrophage (TAM)‐secreted exosomal miR‐29a‐3p. Hypoxic conditions also enhance the proliferation and function of myeloid‐derived suppressor cells (MDSCs) and lead to an immunosuppressive environment. Agents targeting diverse proteins were validated to impede the activation of Wnt pathway, including anti‐Wnt mAbs and small molecular inhibitors of Wnt ligands, low‐density lipoprotein (LDL)‐related protein (LRP) inhibitors, small molecular inhibitors targeting β‐catenin, and tankyrase inhibitors that promote β‐catenin degradation.

The first Wnt gene, *Wnt1*, which accounts for mammary tumorigenesis in mice, was discovered approximately 40 years ago.^34^ At the turn of the century, most of the crucial elements in the Wnt pathway had been discovered. In the canonical Wnt cascade, the ligand‐bound Wnt receptor complexes can block the phosphorylation of β‐catenin. Therefore, the component of specific E3 ubiquitin ligase β‐TrCP can no longer recognize and deregulate β‐catenin. Stabilized β‐catenin binds to T‐cell factor (TCF) in the nucleus, promoting the transcription of Wnt downstream genes. Wnt signaling is crucial for embryonic development and tissue homeostasis in nature. It regulates stem cell self‐renewal and determines the cell fate in various organs, including the intestine and skin, which tumor suppressors tightly control via negative feedback loops or direct regulation.^35^, ^36^, ^37^ However, persistent hyperactivation of the Wnt pathway is observed in various kinds of cancer cells^38^ (Figure 2).

The role of the Wnt pathway in cancer has been extensively elucidated, and its functions primarily include promoting tumor proliferation, metastasis, stem cell maintenance, and drug resistance.^39^ In gastric cancer, long non‐coding RNA (lncRNA) small nucleolar host gene 11 (SNHG11), upregulated in multiple cancer, induced glycogen synthase kinase 3β (GSK‐3β) ubiquitination activate the Wnt/β‐catenin pathway and contribute to cell proliferation, stemness, migration, invasion, and EMT.^40^ In addition to the canonical Wnt/β‐catenin cascade mentioned above, currently, there are two different pathways believed to be activated when Wnt receptors are activated: the planar cell polarity (PCP) pathway and the Wnt/Ca2^+^ pathway. Moreover, the noncanonical Wnt pathways play crucial roles in embryonic development and can also be hijacked and activate the transcription of downstream target genes by tumor cells.^39^ For example, frizzled family receptor 7 (FZD7), a receptor for Wnt signaling, is associated with aggressiveness in Stem‐A ovarian cancer by casein kinase 1ɛ‐mediated non‐canonical Wnt/PCP pathway.^41^

With the continuous development of antitumor drugs, the role of the Wnt pathway in tumor treatment resistance is increasingly emerging. A body of research has shown that the activity of the Wnt/β‐catenin pathway can be upregulated in various cancers,^42^, ^43^, ^44^ confer tumor cells the ability to survive in the insult from radiotherapy or chemotherapeutics, such as 5‐fluorouracil (5‐FU), oxaliplatin, temozolomide, and other agents.^45^, ^46^, ^47^ For instance, chronic hypoxia increased the expression of hypoxia‐inducible factor 2α (HIF‐2α) and induced the resistance of breast cancer cells to paclitaxel (PTX).^48^ More precisely, HIF‐2α overexpression increases the activation of Wnt signaling. Dickkopf‐1, a Wnt inhibitor, strikingly reverses the resistance to PTX.^46^ In addition to chemotherapy, hyperactivation of the Wnt pathway is also associated with decreased sensitivity to radiation therapy. The miR‐301a in hypoxic glioma cell‐derived exosomes can directly target *TCEAL7*, which upregulates the activity of the Wnt pathway by boosting β‐catenin translocation from the cytoplasm into the nucleus and enhances radiation sensitivity. This resistance to radiotherapy can be reversed by inhibiting the activation of the Wnt/β‐catenin pathway.^49^ Moreover, reactivation of Wnt signaling is also involved in resistance to immunotherapy by disrupting various components of tumor immunity.^50^, ^51^, ^52^ In autochthonous mouse melanoma models, active β‐catenin signaling negatively regulates the antitumor T‐cell responses.^53^ This study found that melanoma without T‐cell infiltration was highly linked to tumor‐intrinsic Wnt pathway activation. Meanwhile, active Wnt/β‐catenin cascade contributes to T‐cell exclusion and therefore results in resistance to antiprogrammed death‐ligand 1 (anti‐PD‐L1)/anticytotoxic T lymphocyte antigen 4 immunotherapy.^54^ Tumor‐associated macrophage (TAM)‐derived exosomal miR‐29a‐3p enhances PD‐L1 expression in ovarian cancer, promoting tumor proliferation and immunosuppression. Examination of the underlying mechanism shows that miR‐29a directly targets FOXO3, which results in the inhibition of an antagonist of the Wnt pathway, termed GSK3β, to facilitate Wnt pathway activation and PD‐L1 expression.^55^, ^56^ Similarly, hypoxic conditions also enhance the expression of miR‐29a in glioblastoma (GBM), which can promote the proliferation of myeloid‐derived suppressor cells (MDSCs) and eventually lead to an immunosuppressive environment.^57^, ^58^

### 2.2 Hyperactivation of notch signaling pathway

Notch signaling has been known for over a century, and its roles in embryonic and organ development have been well studied.^59^, ^60^, ^61^ When the classical Notch signaling cascade is activated, three cleavages occur.^62^ In brief, after synthesis in the endoplasmic reticulum, the Notch extracellular domain (NEC) of the receptor can be transferred into the Golgi compartment and then processed by furin‐like convertase (S1 cleavage). At the cell surface, NEC and Notch transmembrane fragment are linked by disulfide bonds to form the heterodimeric Notch receptor, which interacts with its ligand on the juxtaposed cell. With ligand interaction exposed to disintegrin and metalloproteases (ADAM) metalloproteases (S2 cleavage), the C‐terminal cleavage domain of the receptor is further cleaved by the γ‐secretase complex (S3 cleavage). Finally, the liberated Notch intracellular domain (NICD) is translocated to the nucleus and forms a trimeric complex with CSL (also known as CBF1) and mastermind‐like protein (MAML), changing CSL function to initiate its related transcription of downstream targets. The Notch pathway participates in the developmental programs of most organs and tissues and often plays an iterative role during the progression of a particular cell lineage.^62^ Although there are many conditions where the Notch pathway blocks differentiation and secures a pool of stem or progenitor cells (PCs), in some contexts, the Notch pathway can promote differentiated cell fate, for example, in the skin.^63^ Notch pathway is also essential for the formation of lateral inhibition in some differentiation programs, such as in the inner ear development.^64^

**FIGURE 3:**
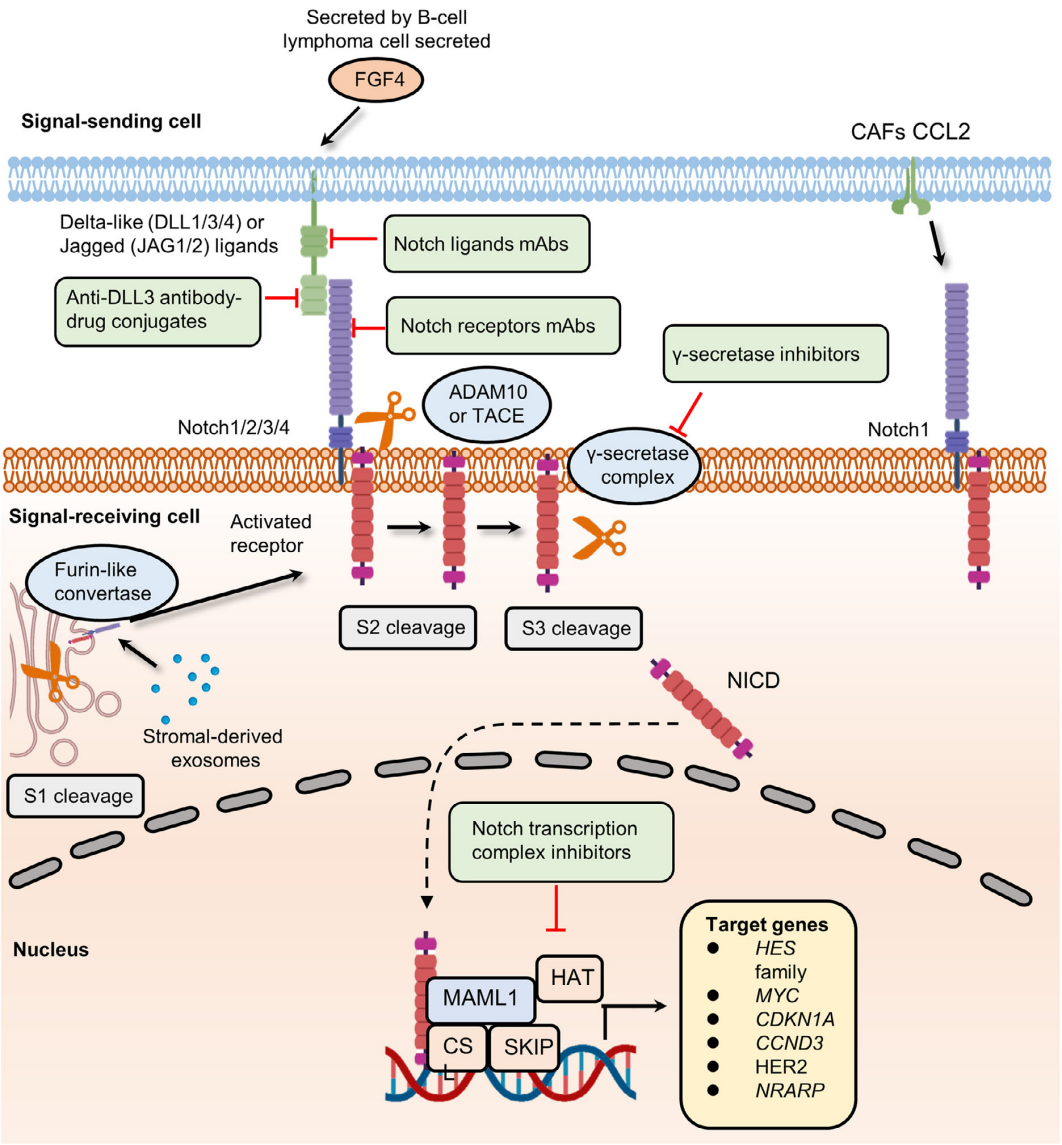
The canonical Notch signaling pathway and related pharmacological inhibitors that reverse Notch pathway‐induced cancer progression. The activation of Notch receptors needs to undergo three times cleavages. The first‐time cleavage, known as S1 cleavage, occurs in the Golgi apparatus and is mediated by a furin‐like convertase. Following S1 cleavage, the interaction between signal‐sending cell Notch ligands (Delta‐like ligands (DLL1, DLL3, and DLL4) and Jagged ligands (JAG1 and JAG2)) and signaling receiving cell Notch receptors (Notch1, Notch2, Notch3, and Notch4) on the cell surface can result in the cleavage of Notch receptors mediated by ADAM10 or ADAM17, termed (S2 cleavage). In the end, γ‐secretase‐mediated S3 cleavage leads to the release of NICD and the formation of Notch transcription complex. The expression of Notch pathway target genes promotes the development of cancer drug resistance. The Notch signaling receptor Notch1 was upregulated by overexpression of chemokine (C‐C motif) ligand 2 (CCL2) in CAFs, and FGF4 in adjacent tumor cells stimulated the expression of Jag1. Both regulatory axis contributed to the activation of the Notch pathway. Moreover, several agents were used to inhibit the activation of Notch signaling compressing Notch ligands mAbs, anti‐DLL3 antibody–drug conjugates, Notch receptors mAbs, γ‐secretase inhibitors, and Notch transcription complex inhibitors.

Notch signaling's dual function in cell fate decisions (blocking or promoting differentiation) under different conditions may endow cancer cells with the ability to promote the development of tumors as well as drug resistance^65^, ^66^, ^67^ (Figure 3). For instance, FGF4 secreted by B‐cell lymphoma cell promotes the expression of Jag1 within endothelial cells, activating Notch2 in adjacent cancer cells. The juxtacrine pathway promotes Notch signaling activation in endothelial cells thereby inducing invasiveness and chemoresistance.^68^ The above results are consistent with previous evidence about the oncogenic role of Jag1 in breast, colon, and liver cancers.^69^, ^70^, ^71^ The upregulation of Notch receptors could also activate the Notch pathway.^72^, ^73^ Stromal‐derived exosomes containing noncoding transcripts and transposable elements can be delivered into breast cancer cells and activate the STAT1‐dependent antiviral pathway.^74^ In turn, active antiviral signaling promotes Notch3 expression and Notch signaling‐dependent therapeutic resistance.^74^ Other important stromal cells involved in upregulating the Notch pathway are cancer‐associated fibroblasts (CAFs).^75^ Primary CAFs enhance the expression of chemokine (C‐C motif) ligand 2 (CCL2), which contributes to the stemness maintenance of breast cancer cells. Increased CCL2 expression is correlated with high expression of Notch1 and therefore confers Notch signaling‐induced CSC features in vitro and in vivo.^76^ Another research revealed that downregulating CCL2 expression could significantly reduce carcinogenesis and Notch1 expression in a xenograft model containing both fibroblasts and breast cancer cells.^77^ Taken together, the data demonstrate that cancer cells can directly or indirectly hijack Notch signaling to promote cancer progression.

### 2.3 High‐level activation of hedgehog signaling

**FIGURE 4:**
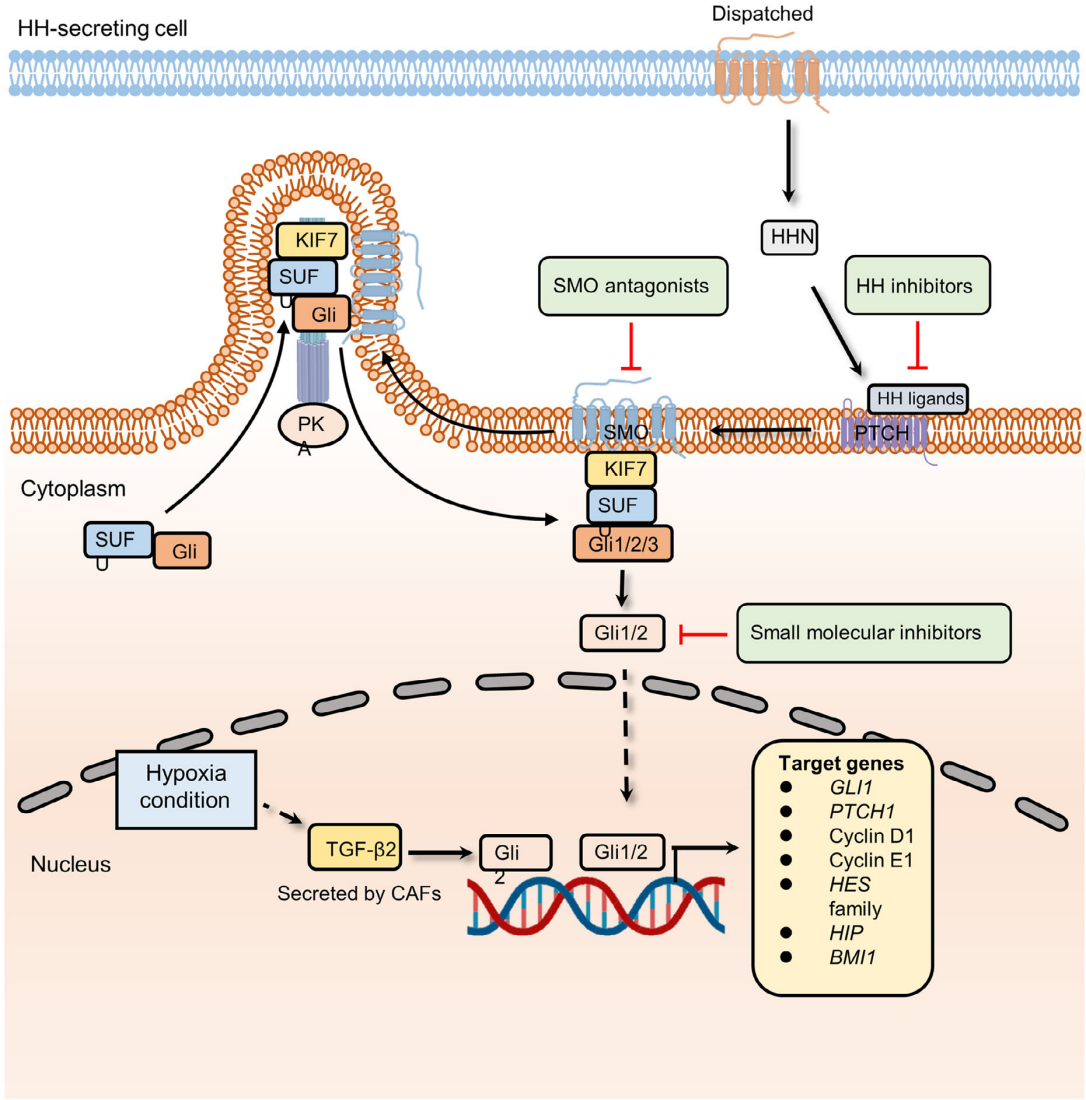
The canonical Hedgehog signaling pathway involved in cancer and associated therapeutic targets. The secretion of HHN regulated by Dispatched homolog can bind to PTCH receptor and hence releases SMO. Then, accumulated SMO, sequestrated kinesin family member 7 (KIF7), suppressor of fused homolog (SUFU) proteins, and Gli transcription factors form a multiprotein complex in cilia, which prevents Gli from inhibitory phosphorylation by PKA. Stable Gli is further released from SUFU complex and mediates the transcription of Hedgehog signaling pathway. CAF‐derived hypoxia stimulates the expression of TGF‐β2, which increases the level of Gli2 and activates Hedgehog signaling. Meanwhile, SMO antagonists, HH inhibitors, and small molecular inhibitors were proven to inhibit hedgehog pathway activation.

Hedgehog signaling is a vital pathway that determines cell location and fate in early embryonic development.^78^, ^79^, ^80^ Following the development, the Hedgehog pathway participates in tissue homeostasis and wound healing.^81^, ^82^, ^83^ The canonical mammalian Hedgehog signaling cascade can be activated by the interaction between Hedgehog ligands (Desert Hedgehog, Indian Hedgehog, and Sonic Hedgehog (SHH)) and Hedgehog receptors (Patched‐1 (PTCH1) and Patched‐2 (PTCH2)).^84^ Their interplay results in the phosphorylation of Smoothened (SMO), the main effector of Hedgehog signaling, and the inhibition of multiprotein complexes containing GSK3β, protein kinase A (PKA), and suppressor of fused homolog (SUFU). The proteolysis of transcription factor glioma‐associated oncogene family zinc finger (Gli) is then blocked. Finally, Gli transcription factors translocate to the nucleus and activate transcription at start sites. During embryonic development and tissue homeostasis, Hedgehog signaling is typically modulated spatially and temporally. Given the important roles of Hh pathway in in the maintenance of stem PCs in many adult tissues, dysregulation of Hedgehog signaling can drive the development of several cancers, such as basal cell and colorectal carcinomas^85^, ^86^, ^87^ (Figure 4).

As the most common skin cancer in the western world, basal cell carcinoma (BCC) was first linked to BCC through the identification of germline mutations in Ptch1, which are responsible for Gorlin syndrome (also known as nevoid BCC syndrome or NBCCS).^88^ Lineage tracing experiments showed that activating Smo oncogenes in interfollicular epidermal stem cells (IFE‐SCs), but not in hair follicle bulge stem cells, led to BCC development. This pinpointed IFE‐SCs as the source of BCC in mice.^89^ Subsequent investigations showed that Smo activation in IFE‐SCs resulted in more aggressive tumor growth compared to Smo activation in PCs. This heightened growth was attributed to the greater capacity of SCs for symmetric self‐renewing divisions and their increased P53‐dependent resistance to cell death compared with PCs.^90^ As expected, given the crucial role of the Hh signaling pathway in maintaining stemness, its role in tumor treatment resistance is continuously being unveiled. A multidimensional genomics analysis revealed that the active transcription factor serum response factor (SRF) could cause Gli1 transcriptional activity amplification in drug‐resistant BCCs.^87^ The overexpression of Gli1 confers the activation of the Hedgehog pathway as well as drug resistance.^91^, ^92^ A recent study indicates that CAFs and hypoxia are involved in chemoresistance by upregulating the expression of Gli2 (a ligand of Hedgehog signaling).^86^ In this study, researchers found that low‐oxygen conditions could induce CAFs to secrete TGF‐β2. High‐level TGF‐β2 augments the transcription of Gli2, promoting the occurrence of drug resistance. Moreover, a retinoic acid‐low (RA‐low) microenvironment plays a crucial role in bortezomib (BTZ) resistance.^93^ Multiple myeloma cells secrete paracrine Hedgehog, which increases the expression of stromal CYP26, a cytochrome P450 monooxygenase, favoring the establishment of an RA‐low microenvironment. Inhibition of retinoid signaling blocks the differentiation of malignant hematopoietic cells and reduces BTZ sensitivity.^93^ In addition, endothelial cells can promote the stem‐like phenotype of cancer cells by activating the Hedgehog pathway.^94^ In glioma cells, the expression of stemness‐related genes, including *Sox2, Olig2, Bmi1*, and *CD133*, was upregulated when cocultured with endothelial cells. However, knockdown of Smo in endothelial cells abolished the stem‐like phenotype in glioma cells. To further unveil the mechanism of the activation of the Hedgehog signaling cascade in perivascular glioma cells, tissue specimens from glioma patients were examined, which indicated that some canonical development pathways, such as the Wnt pathway, could be the “intermediary” to promote Hedgehog signaling‐mediated drug resistance. In a study combining a 3D culture model and whole‐transcriptome analysis, Wnt and Hedgehog signaling components were found to be overexpressed in CRC.^95^ Of note, Wnt signaling is negatively regulated by the canonical Gli‐dependent Hedgehog pathway in CRC. To further investigate the underlying mechanism, the expression of specific Hedgehog pathway genes was detected. The results showed that the activation is mainly driven by Gli‐independent and noncanonical Hedgehog signaling components, which are the positive regulators of the Wnt pathway. Noncanonical Hedgehog signaling cooperates with Wnt pathway to maintain the stemness of CSCs and develop resistance to antitumor drugs.^96^

### 2.4 Aberrant regulation of hippo signaling cascade

**FIGURE 5:**
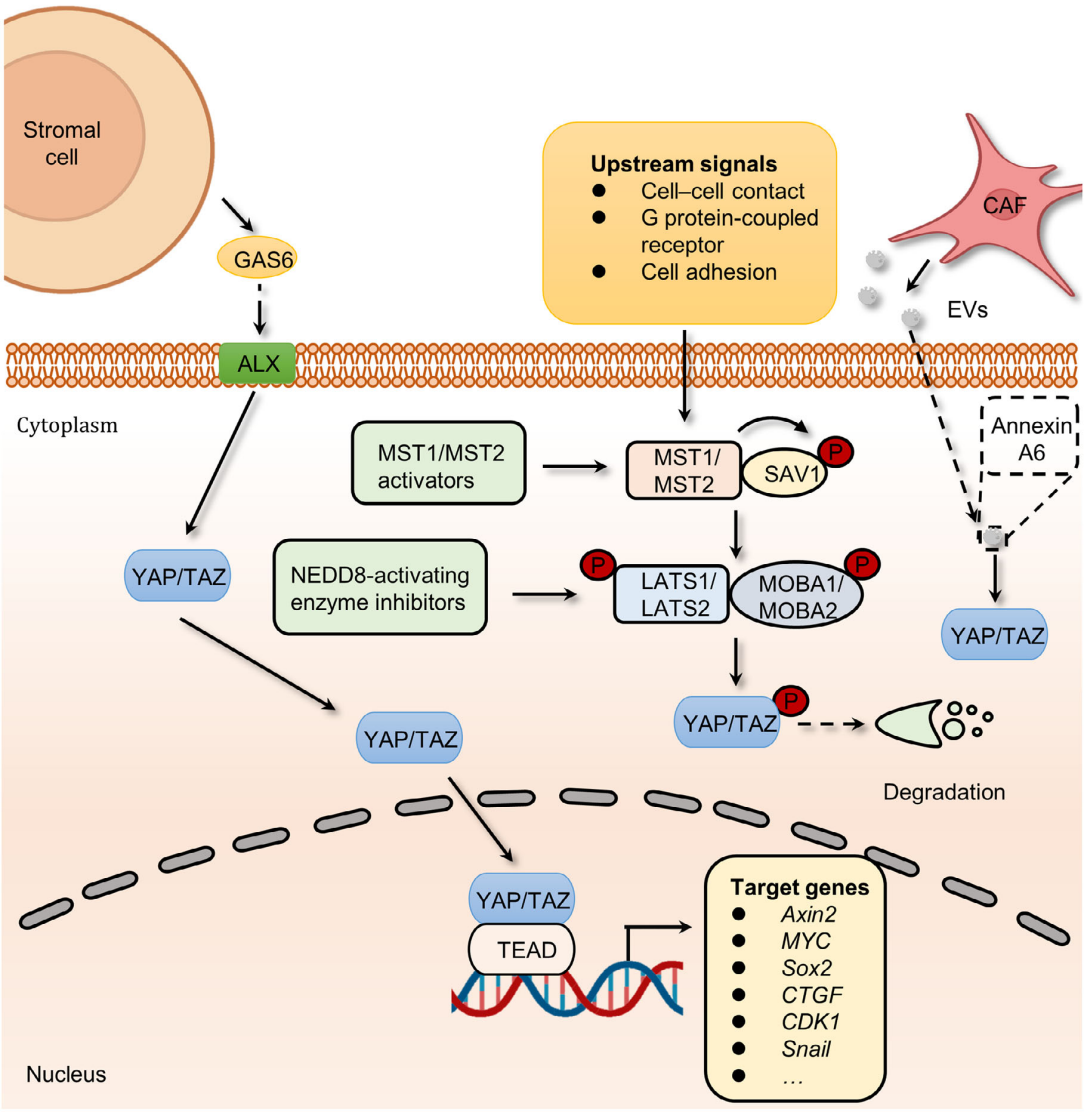
The mechanism by which cancer cells utilize the Hippo signaling pathway and associated therapeutic targets. The core proteins of Hippo pathway include MST1/2, LATS1/2, SAV1, MOB1A/1B, and YAP1/TAZ. Once activated by upstream signals, MST1/2 can phosphorylates SAV1, which subsequently activates LATS complex comprising LATS1/2 and MOB1A/1B. Then, the activated LATS complex phosphorylates YAP1/TAZ, thereby leading to the degradation of transcriptional factors of Hippo pathway. Otherwise, YAP1/TAZ can translocate to the nucleus and bind with TEA domain (TEAD) family members to promote the expression of Hippo signaling target genes. Stromal cells‐secreted GAS6, and CAF‐derived EVs (encompassing Annexin A6) mediate YAP/TAZ shuttling to the nucleus, which favors the expression of drug resistance‐related genes. MST1/2 activators and NEDD8 activating enzyme inhibitors were developed to prevent the transcription of Hippo pathway target genes, thereby increasing the degradation of YAP1/TAZ.

Hippo signaling plays an important role in mediating organ development, tissue homeostasis, immune modulation, and wound healing.^97^, ^98^, ^99^, ^100^ At the turn of the 21st century, Hippo pathway was found to restrict the growth of Drosophila tissues.^101^ In the mammalian canonical Hippo kinase cascade, the MST1/2–Salvador homolog 1 (SAV1) complex phosphorylates and activates LATS1/2–MOB domain kinase activator 1A/1B (MOB1A/1B) complexes. The activated LATS1/2–MOB1A/B complex then phosphorylates and inactivates YAP/TAZ, thereby inhibiting the transcription of downstream target genes^102^ (Figure 5).

Analyses of various tumors and cancer cell lines, as well as data from The Cancer Genome Atlas with over 9000 tumors, emphasize the prominent role of YAP/TAZ in cancer, with the Hippo pathway being one of the frequently altered signaling pathways in human cancer.^103^ Remarkably, the YAP1 and WWTR1 genes, which code for YAP and TAZ respectively, undergo amplification in approximately 14% of head and neck squamous cell carcinomas (HNSCCs), around 16% of lung squamous carcinomas, approximately 17% of cervical squamous cell carcinomas, and about 15% of esophageal squamous cell carcinomas.^103^ As mentioned above, the Hippo pathway can promote the occurrence and development of tumors by regulating processes such as tumor cell migration and immune modulation. Initial investigations demonstrated that the ectopic expression of YAP, particularly nuclear‐localized mutants of YAP, exhibits robust prometastatic activity. This activity is contingent on TEAD binding, implying that interfering with this interaction might hold therapeutic promise in aggressive cancers. YAP further facilitates a metabolic shift in cancers with lymph node metastasis by stimulating the expression of genes that bolster fatty acid oxidation.^104^ In addition, both YAP and TAZ have the ability to induce the expression of programmed cell death 1 ligand (PD‐L1), a ligand for the programmed cell death‐1 (PD‐1) receptor, which in turn creates an immunosuppressive microenvironment.^105^ TAZ, for instance, stimulates the expression of PD‐L1 in lung and breast cancer cells, while YAP promotes PD‐L1 expression in melanomas and HNSCC cells.^106^ Consequently, targeting YAP/TAZ presents an appealing strategy to reduce PD‐L1 levels and, as a result, enhance the efficacy of immunotherapy employing neutralizing antibodies against PD‐L1 and/or PD‐1. Meanwhile, YAP/TAZ have been found to participate in various Hippo pathway‐dependent drug resistance and show a central role in mediating resistance to cancer therapeutics.^107^, ^108^, ^109^ For instance, extracellular vesicles (EVs) from CAFs contribute to the activation of focal adhesion kinase (FAK)‐YAP signaling and enhance drug resistance.^110^ In this work, a comprehensive proteomic analysis of CAF‐EVs was performed, which identified a key effector, Annexin A6, involved in drug resistance. Annexin A6 secreted by CAFs activated FAK‐YAP by stabilizing β1 integrin at the cell surface of gastric cancer cells and in turn resulted in resistance to cisplatin. More recently another study also discovered that CAFs within gastric cancers expand resistance to 5‐FU by activating YAP/TAZ.^111^ In addition to chemotherapy, the inactivation of Hippo signaling confers resistance to targeted therapies. For example, the links between the activation of YAP/TAZ and tyrosine kinase inhibitors (TKIs) resistance have been revealed.^112^, ^113^ Although the precise mechanism of how YAP/TAZ signaling develops resistance to TKIs is poorly understood, AXL, one of the YAP/TAZ transcriptional targets, seems to be a candidate to drive resistance.^114^, ^115^ AXL is a receptor tyrosine kinase that belongs to the Tyro3, Axl, and Mer (TAM) receptors and is activated by growth arrest‐specific protein 6 (GAS6).^116^, ^117^ At present, hyperactivation of GAS6/AXL signaling is considered as one of the hallmarks in many types of multidrug‐resistant cancer cells.^118^, ^119^, ^120^, ^121^, ^122^ Interestingly, a study revealed that stromal cells in the tumor microenvironment (TME) could continuously express GAS6 to activate the AXL receptor of adjacent tumor cells, thus promoting cancer drug resistance.^123^ The administration of AXL inhibitors, such as R428, abolishes GAS6/AXL signaling‐induced resistance to quizartinib.^119^ Taken together, the above studies present concrete proof for the key roles of Hippo signaling in the development of cancer and drug resistance.

### 2.5 Abnormal activation of other signaling pathways

In addition to the above‐mentioned embryonic development‐related pathways, the TGF‐β superfamily and FGF/FGFR signaling cascades also contribute to cancer development and progression.

**FIGURE 6:**
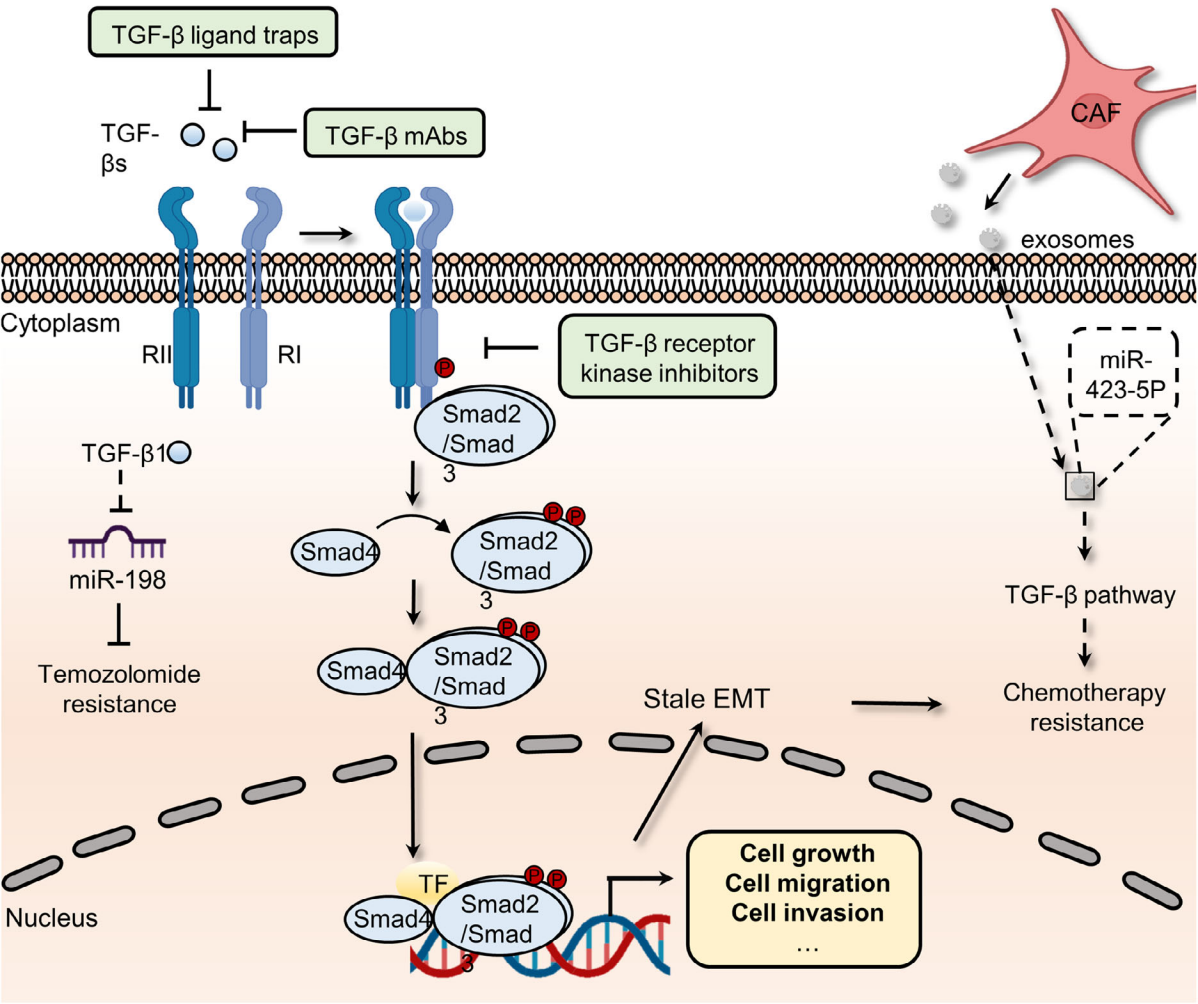
The roles of canonical TGF‐β signaling pathway in cancer and inhibitors that target TGF‐β pathway. The ligands, TGF‐βs, bind TGF‐β type II (RII) induce the phosphorylation of TGF‐β type I (RI) receptors on cell surface. Activated kinase activity of RI further phosphorylates Smad2 and Smad3, which form trimeric complexes with Smad4. These Smad complexes will translocate into nucleus and promote the transcription of TGF‐β pathway downstream genes. Sustained activation of TGF‐β signaling can lead to cancer drug resistance via inducing stable epithelial‐to‐mesenchymal transition (EMT) and regulating the expression of miR‐198 or miR423‐5p. Hence, agents, such as TGF‐β ligand traps, TGF‐β mAbs, and TGF‐β kinase inhibitors, have been developed to impair the activation of TGF‐β signaling pathway.

TGF‐β superfamily signaling, which contains over 30 ligands, including TGF‐βs, growth and differentiation factors, bone morphogenetic proteins, Nodal, and Activins, is required for the development and homeostasis of complex multicellular animals.^124^ These members and their downstream pathway components are highly conserved during evolution and contribute to various cellular functions, such as migration, differentiation, growth, apoptosis, and adhesion.^124^ To perform these complex biological functions, ligand dimers need to bind to and activate heterogeneous complexes of type I and type II receptors that phosphorylate intracellular mediators (Smads). Then, phosphorylated mediators form complexes with each other and other components to mediate the transcription of target genes in the nucleus.^125^ Finally, the expression of effectors leads to related cellular responses during different embryonic developmental stages. For instance, TGF‐β family members can induce EMT, an essential process in the temporally distinct phases of heart development, by upregulating the expression of related markers, including Snail1/2, ZEB1/2, Twist, and ids.^125^ TGF‐β signaling dysregulation, on the other hand, may drive cancer cell proliferation, metastasis, and drug resistance (Figure 6). For example, mutations in the SMAD gene are detected in 60% of pancreatic cancer patients. The coexistence of KRAS mutations and Smad mutations in pancreatic ductal adenocarcinoma (PDAC) patients plays a crucial role in driving early tumor formation and metastasis.^126^ Through Smad signaling, TGF‐β1 suppresses the expression of miR‐198, a cellular methylguanine DNA methyltransferase regulator, in GBM to alter temozolomide sensitivity.^127^ Meanwhile, CAF‐secreted exosomal miR‐423‐5p promotes chemotherapy resistance by regulating the TGF‐β pathway.^128^ The TGF‐β pathway in prostate cancer cells is activated by miR‐423‐5p, which leads to the inhibition of GREM2, a differential screening‐selected gene aberrant in the neuroblastoma (DAN) family member, and enhances drug sensitivity. Of note, current studies reported the vital function of TGF‐β in attenuating TME response to PD‐L1 blockade. The role of the TGF‐β pathway in restraining antitumor immunity by restricting T‐cell infiltration was revealed by examining tumors from a large cohort of patients with metastatic urothelial cancer treated with atezolizumab, a PD‐L1 inhibitor, and using a mouse model to recapitulate the above findings.^129^ Therapeutic cotreatment with TGF‐β blockage and anti‐PD‐L1 antibodies reverses TGF‐β signaling‐induced T‐cell exclusion in the center of tumors, which triggers robust antitumor immunity and tumor regression. Moreover, YM101, an anti‐TGF‐β/PD‐L1 bispecific antibody, was also found to reshape the immunosuppressive microenvironment induced by the TGF‐β/Smad pathway and promote the formation of “hot tumors”.^130^ In addition, TGF‐β signaling fosters drug resistance and regulates stemness in various cancers. For example, compared to the reversible state induced by a shorter exposure, chronic TGF‐β exposure could drive stable EMT, tumor stemness, and chemoresistance in breast cancer cells.^131^ Similarly, informative research showed that HIF‐1α and CAF‐secreted TGF‐β2 converge to enhance the expression of Gli2 in CSCs, promoting stemness and resistance to chemotherapy.^86^

FGF/FGFR pathway is essential for early development of embryonic tissue or organ (such as the skeleton, lung, urinary system, and heart).^132^ As one of the most diverse growth factor groups in vertebrates, the FGF family regulates lots of functions, comprising survival, proliferation, differentiation, and migration.^132^ At present, 22 FGF ligands have been discovered in mice and humans that exert their pleiotropic effects through binding high‐affinity tyrosine kinase receptors, including FGFR1, FGFR2, FGFR3, FGFR4, and FGFRL1.^133^ The binding of FGFs to FGFRs triggers conformational changes and the phosphorylation of tyrosine residues within the cytosolic tail of FGFRs, leading to dimerization and activation of cytosolic tyrosine kinases.^134^ Then, the phosphorylated tyrosine residues provide docking sites for downstream signaling molecules and subsequently regulate their related pathways, including MAPK, PI3K/AKT, and STAT signaling.^134^ In addition to embryonic development, accumulating evidence has revealed the significant functions of the FGF/FGFR pathway in the development of cancer. The overexpression of FGFs and FGFRs in cancer cells has been related to a poorer prognosis in a growing number of studies.^134^ Moreover, FGF/FGFR axis‐dependent downstream signaling cascades play indispensable functions in this process. A recent research reveals the presence of key FGFR components in cervical cancer cell lines and their potential role in promoting invasive disease characteristics, highlighting the potential for therapeutic interventions targeting FGFR in cervical cancer treatment.^135^ In HNSCC cells, FGFR3 overexpression activated MAPK signaling and upregulated the level of ERK, which in turn boosted FGF2 production and resistance to bevacizumab.^136^ Similarly, the PI3K/AKT pathway was also identified as an important mediator in FGF/FGFR‐dependent cancer drug resistance. Overexpression of FGFR1 could increase AKT activation, leading to EMT and resistance to the first‐line EGFR inhibitor gefitinib in non‐small cell lung cancer (NSCLC).^137^ Another downstream signaling pathway of the FGF/FGFR pathway involved in cancer is STAT signaling. In breast cancer cells, FGFR1 was shown to promote the synthesis of hyaluronan by activating STAT3 signaling. Blocking either hyaluronan synthesis or STAT3 activation reverses proliferation and doxorubicin resistance of breast cancer cells.^138^

## 3 DORMANT CANCER CELL RESEMBLING EMBRYONIC DIAPAUSE

Insects utilize diapause in response to harsh environments (such as cold and nutrient deficiency).^139^, ^140^, ^141^ Similarly, a large number of mammalian species, including mice, kangaroos, and deer, can also delay blastocyst implantation until they meet suitable conditions.^142^, ^143^, ^144^, ^145^ Before pregnancy, the blastocysts severely decrease their metabolic rate and block cell division for up to one year to prepare for future implantation.^146^, ^147^ Accordingly, dormant cancer cells also were found to enter an embryonic diapause‐like state following treatment with antitumor medications.^148^, ^149^, ^150^

### 3.1 The definition of dormant cancer cell

Ever since Willis initially coined the term, “dormancy” has taken on varying interpretations among researchers, leading to potential misunderstandings, especially for individuals not directly engaged in this field. In clinical practice, the term “dormancy” is employed to describe the extended interval between primary tumor treatment and the recurrence of metastases in secondary locations.^151^ Although dormant tumor cells and tumor stem cells share many similarities, such as drug resistance and their critical roles in recurrence, there are also significant differences between the two concepts. To begin with, there is no direct experimental evidence indicating that CSCs have experienced cell cycle arrest. Furthermore, not all dormant tumor cells can be detected with the same stemness markers as CSCs, such as SRY‐box 2 (SOX2) and Nanog Homeobox (NANOG).^152^

Dormant cancer cells are a specific population that displays reversible cell cycle arrest and acquires the abilities to gain additional mutations, adapt to new environments and drive cancer drug resistance.^153^, ^154^, ^155^ Conversely, extrinsic environmental signals (e.g., cell–matrix interface or chronic inflammation) and cellular regulatory mechanisms (e.g., the upregulation of Myc) are able to awaken quiescent tumor cells to re‐enter the proliferative cycle.^156^, ^157^, ^158^, ^159^, ^160^ Presently, dormant cells have been found in several malignancies, including breast, colorectal, pancreatic, and ovarian cancers, acute myeloid leukemia, and GBM, and this rare subpopulation is thought to be responsible for lesions relapse.^152^, ^161^, ^162^, ^163^, ^164^, ^165^, ^166^ However, key mechanisms, such as how dormant cells utilize their specific state to adapt to new ecological niches, resist initial drug assaults, and transit between dormancy and activation states, have been poorly identified.^151^

### 3.2 Dormant cancer cells and cancer progression

**FIGURE 7:**
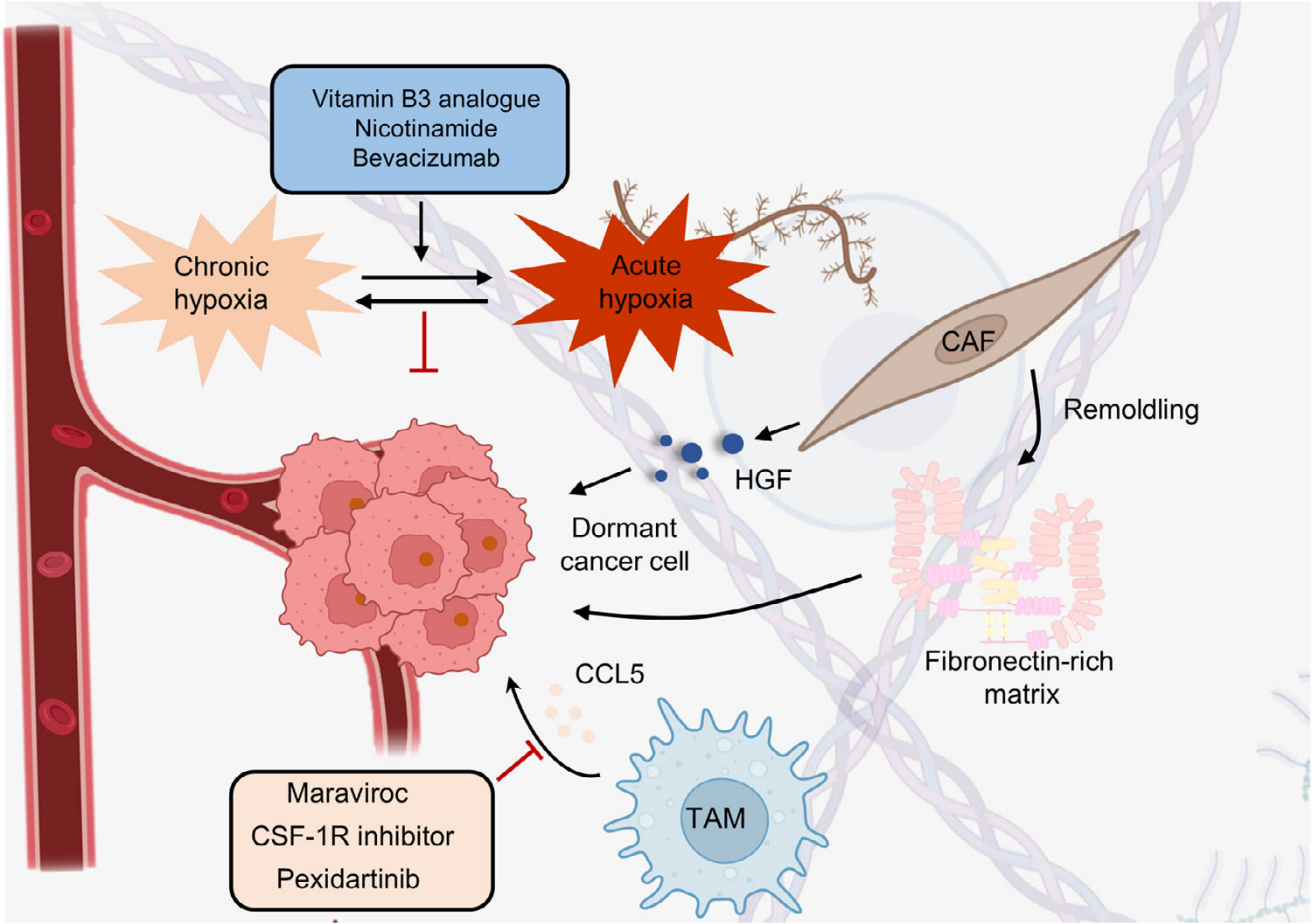
The important roles of the niche in dormant cancer cells. Tumor recurrence is closely related to dormant cancer cells, and the maintenance of dormant cells is significantly associated with the TME (such as the hypoxic microenvironment, CAFs, and TAMs). CAFs favor dormant cancer cells by secreting HGF or stimulating the remodeling of the fibronectin‐rich matrix. By altering the TME, the dormancy of tumor cells can be activated, and the recurrence of lesions could be inhibited. Vitamin B3 analogs, nicotinamide, and bevacizumab could augment radiotherapy by changing the oxygen content in the TME. Meanwhile, by applying maraviroc, CSF‐1R, and pexidartinib, the recruitment of TAMs could be abolished, enhancing therapeutic outcomes in various cancers.

Most theories agree that dormant cancer cells are caused by genetic variants rather than nongenetic variants.^167^, ^168^, ^169^ Moreover, whole‐exome and whole‐genome sequencing studies showed little difference in the mutational landscape between primary and metastatic cancer cells (arising from dormant cells).^170^, ^171^, ^172^, ^173^, ^174^ Is it possible that niches can be reprogrammed to induce alterations within dormant cells? In support of this, a recent study indicated that stromal changes in aged lungs result in the occurrence of melanoma dormancy.^175^ The role of lung aged fibroblasts in inducing the transition from dormant phenotype to outgrowth was revealed by intradermally injecting melanoma cells into young or old C57BL6 mice, implying the niche dependence of dormant cells. Meanwhile, dormant cancer cells within BRAF^V600E^‐mutated mice and human melanoma are tightly linked with the activation of CAFs. BRAF inhibition‐induced activation of CAFs stimulates the remodeling of the fibronectin‐rich matrix, consequently contributing to melanoma cell persistence by the reactivation of ERK.^176^ Moreover, CAFs are internalized and degraded by breast cancer cell cannibalism.^177^ The modulation of cell cannibalism is determined by Jun N‐terminal kinase, EMT, and stem cell‐like markers, which lead to the activation of deceleration programs and drug resistance in vitro.^178^, ^179^, ^180^ In addition, CAF‐derived secretory proteins, such as hepatocyte growth factor (HGF), the ligand of MET, trigger the activation of the PI3K–AKT signaling pathway.^181^, ^182^, ^183^ This pathway endows resistance to BRAF inhibition in melanoma, colorectal, and glial tumor cells.^184^, ^185^, ^186^, ^187^ In basal‐like HER2^+^ breast cancer, CAF‐secreted HGF also promotes resistance to HER2 inhibitor.^188^ A recent study proposed that the TME could drive cell state transition and drug response in pancreatic cancer.^189^, ^190^ Combining systematic profiling of metastatic pancreatic cancer biopsies and matched organoid models, the functions of TME in modulating the cell state to impact drug response were illustrated. Transcriptional state representation of pancreatic cancer shows strong culture‐specific biases under stimulation with different conditions, highlighting the crucial functions of the niche in regulating cell state (Figure 7).

The above evidence supports the opinion that dormant cancer cells need complex dynamic interactions with different cell types in the niche.^178^, ^191^, ^192^ Therefore, we wondered whether environmental factors could also cause the cell cycle arrest of dormant cells. Physical factors in the niche, such as hypoxia, may inhibit cell proliferation and thus maintain a state of dormancy.^193^, ^194^, ^195^ Indeed, breast cancer cells cultured in chronic intermittent conditions in vitro have been shown to become dormant, characterized by G0‐G1 cell cycle arrest, and hypoxia also results in dormancy in vivo.^196^, ^197^ Moreover, in prostate and breast cancers, crosstalk between cells and their secreted molecules leads to the inhibition of proliferation. For instance, endothelial cell‐secreted thrombospondin 1 has been proven to induce breast cancer cells to exit the cell cycle.^198^, ^199^ In addition to the endothelial niche, the endosteal niche has been shown to regulate the proliferation of multiple myeloma cells and acute lymphoblastic leukemia (ALL).^200^ Coculturing with MC3T3 osteoblast precursor cells and primary osteoblasts conditioned medium can suppress the proliferation of 5TGM1 multiple myeloma cells in vitro.^201^ In this regard, extracellular osteopontin in the endosteal niche can also promote ALL cell cycle arrest and dormant transit.^202^

It is clear that the transition from cancer cells to dormant cancer cells is a major challenge in clinical practice.^203^, ^204^ This transition results in functional alteration of tumor cells to escape damage from chemotherapy drugs as well as immunosurveillance.^205^, ^206^, ^207^ Most antitumor drugs are designed to target cancer cells with high proliferation features and neglect slow‐cycling cells that cause lesion relapse.^208^, ^209^ Several core driver genes and pathways have been reported in this process, including Myc, mTOR, GPX4, Mex3a, oxidative phosphorylation and LINE‐1 repression in 2D or 3D cell models.^210^, ^211^, ^212^, ^213^ Once antitumor drugs are removed, these cells exit dormancy by controlling notable vital factors, such as reactivation of Myc, and grow into a population.^214^, ^215^, ^216^ Finally, these clones provide an opportunity to gain resistance mutations and induce drug resistance.^217^ It is well explained why ALL patients benefit from long‐term oral low‐dose chemotherapy from the end of intensive chemotherapy, even in chemosensitive ALL subtypes.^218^

Regarding how dormant cells evade the surveillance of the immune system, a body of data indicates that this process could be achieved by repressing endogenous antigen presentation, such as major histocompatibility complex class I (MHC I).^219^, ^220^ Indeed, the expression of tumor antigens and MHC I is frequently deficient in individual metastatic cancer cells.^221^, ^222^, ^223^ Moreover, another mechanism by which dormant cancer cells avoid T‐cell recognition and elimination was unveiled in PDAC, whereby tumor cells in the liver that lacked expression of tumor antigen cytokeratin 19 and MHC I selectively responded to endoplasmic reticulum stress.^224^ In contrast to MHC I, MHC II expression may contribute to immunosuppression in patients with melanoma and lung cancer.^225^, ^226^, ^227^ Dormant cancer cells exposed to interferon in the niche could drive the expression of MHC II and other cell surface molecules linked to myeloid‐lineage cells. As a result, myeloma cells are mistaken for niche‐specific local immune cells and shield themselves from immune clearance. Dormant myeloma cells are recognized as myeloid cells, such as osteal macrophages and CD169^+^ bone marrow macrophages, and evade immune surveillance by this mechanism.^228^, ^229^, ^230^

These findings show that the entire life cycle of dormant cancer cells, from quiescence to reactivation, results from interaction with the local niches. Furthermore, the life cycle of dormant cancer cells, accompanied by cell‐intrinsic and cell‐extrinsic control, can be divided into five stages: niche occupancy, niche interaction and engagement, cellular reprogramming for adaption to the niche, long‐term dormancy, and relapse. In this regard, further advances are needed to comprehensively understand the recognition of cell‐extrinsic control of dormant state via the niche.

## 4 ONCOFETAL REPROGRAMMING OF TME

The ability of tumor cells to avoid immunotherapy by altering the microenvironment is similar to embryo implantation, which can maintain an active state of maternal immune tolerance through CD4^+^ regulatory T cells (Tregs).^231^ For example, to extensively characterize the cellular landscape of the human liver from development to disease, single‐cell RNA (scRNA) sequencing was employed, which revealed remarkable fetal‐like reprogramming of the TME.^232^ Specifically, the results showed that the hepatocellular carcinoma (HCC) ecosystem displayed characteristics reminiscent of fetal development, including re‐emergence of fetal‐associated endothelial cells (PLVAP/VEGFR2) and fetal‐like (FOLR2) TAMs. In addition, the distinct roles of NK cells in the initiation and resolution of inflammation in different phases of pregnancy also indicate the plasticity of the fetal immune microenvironment.^233^ More importantly, this fetal‐like feature has been observed in cancer cells, emphasizing the link between embryogenesis and cancer development and progression. This section focuses primarily on TAMs and MDSCs and their roles in regulating TME.

### 4.1 Tumor‐associated macrophages

TAMs have long been recognized to exert crucial roles in immunosuppression, and the increased abundance of TAMs is associated with a worse prognosis for cancer patients.^234^ In the primary tumor environment, TAMs enhancing tumor cell invasion, intravasation, and the viability of tumor stem cells.^235^ Numerous experiments have provided detailed insights into the mechanisms through which TAMs facilitate tumor cell migration and invasion. For instance, in the RIP tag model of pancreatic islet cancer, tumor cells capable of synthesizing IL‐4 can induce TAMs to produce cathepsin proteases B and S. These proteases play a role in degrading and remodeling the extracellular matrix, thereby facilitating the detachment of tumor cells from the tumor.^236^ Meanwhile, in mouse models of breast cancer, TAMs play a significant role in sustaining the survival of CSCs through juxtacrine signaling,^237^ and in HCC mouse models, they achieve this through signaling mediated by TGF β‐1.^238^ At metastatic sites, macrophages associated with metastasis support processes such as extravasation, ensuring tumor cell survival and sustained growth.^235^ Interestingly, in certain contexts, they can also be involved in maintaining tumor cell dormancy.^239^ In both primary and metastatic locations, TAMs exert suppressive effects on the activities of cytotoxic T cells and natural killer cells, which possess the potential to eliminate tumors.

TAMs also play essential roles in many types of ICB‐resistant cancer cells. In lung cancer, P2X7, a crucial sensor of extracellular ATP, is highly expressed in immunosuppressive cells such as TAMs.^240^ TAMs that highly express P2X7 promote “M2‐like” polarization by downregulating STAT6 and IRF4 phosphorylation in vivo and in vitro. Meanwhile, the P2X7‐expressing TAMs in lung cancer are associated with anti‐PD‐1 antibody resistance, which can be overcome by P2X7 inhibitors O‐ATP, A‐740003, and A‐438079 hydrochloride. TAMs were also reported to confer anti‐PD‐1 resistance by expressing c‐Maf.^241^ The inhibition of c‐Maf partly overcomes resistance to anti‐PD‐1 therapy in a subcutaneous LLC tumor model. Furthermore, in an experimental model of melanoma, CD163‐expressing TAMs specifically maintain immune suppression to resist anti‐PD‐1 checkpoint therapy.^242^ These findings highlight the heterogeneity of the TME and the numerous roles of TAMs in regulating tumor progression.

### 4.2 Myeloid‐derived suppressor cells

Based on their density, morphology, and phenotype, MDSCs primarily fall into two subsets: polymorphonuclear (PMN)‐MDSCs and monocytic (M)‐MDSCs. Initially, PMN‐MDSCs were referred to as granulocytic (G)‐MDSCs, but gradually, the term PMN‐MDSCs became more widely adopted due to distinguishing features in morphology and phenotype compared with steady‐state neutrophils. These features include altered buoyancy, reduced granules, decreased expression of CD16 and CD62L, and upregulated CD11b and CD66b.^243^ Furthermore, a unique population of fibrocystic MDSCs (F‐MDSCs) has been identified and characterized in humans.^244^ MDSCs are a heterogeneous population of immature myeloid cells that can inhibit T‐cell and NK‐cell activity and govern tumor growth, premetastatic niche development, and immunotherapy resistance.^245^ In mouse tumor models, tumor‐infiltrating M‐MDSCs were shown to promote an EMT/CSC phenotype, aiding the dissemination of tumor cells from primary sites. Conversely, PMN‐MDSCs infiltrating the lungs supported metastatic tumor growth by reversing the EMT/CSC phenotype and promoting tumor cell proliferation.^246^ In several mouse tumor models, the inhibition of S100A8/A9, the regulatory factors of MDSCs, has been shown to restrict tumor growth by diminishing the accumulation of MDSCs.^247^ A recent study revealed that the number of MDSCs is related to the antitumor immune response induced by a PD‐1 antibody in mouse models of gastric cancer.^248^ 5‐FU can increase the effects of anti‐PD‐1 by reducing the number of MDSCs. In brief, PD‐L1 expressed by gastric epithelial cells increases the accumulation of MDSCs, which promotes tumor growth and worsens the immune response to PD‐1. Furthermore, MDSCs are involved in KRAS‐interferon regulatory factor 2 (IRF2) axis‐induced immunotherapy resistance in CRC.^249^ Oncogenic KRAS^G12D^ can suppress the expression of IRF2, which leads to direct inhibition of CXCL3 expression. High expression of CXCL3 binds to CXCR2 on MDSCs and promotes the development of immune therapy resistance. Another component within cancer cells that mediates reciprocal communication between tumor cells and MDSCs is cell cycle‐related kinase (CCRK).^250^ Simultaneous overexpression of CCRK and MDSC markers (CD11b/CD33) in HCC significantly reduces the efficacy of immunotherapy. Mechanistically, hepatic CCRK activates the nuclear factor‐κB (NF‐κB)/IL‐6 axis, resulting in the accumulation of (PMN)‐MDSCs resistant to PD‐L1 blockade. Apart from the above conditions, MDSCs have been proven to decrease the efficacy of PD‐L1 blockade in many kinds of cancers, including lung cancer, pancreatic cancer, and melanoma, highlighting the potential of targeting MDSCs for reversing resistance to immunotherapy.^251^, ^252^, ^253^

## 5 OVEREXPRESSION OF EMBRYO/FETAL TRANSPORTERS HAMPERS CANCER THERAPY

**FIGURE 8:**
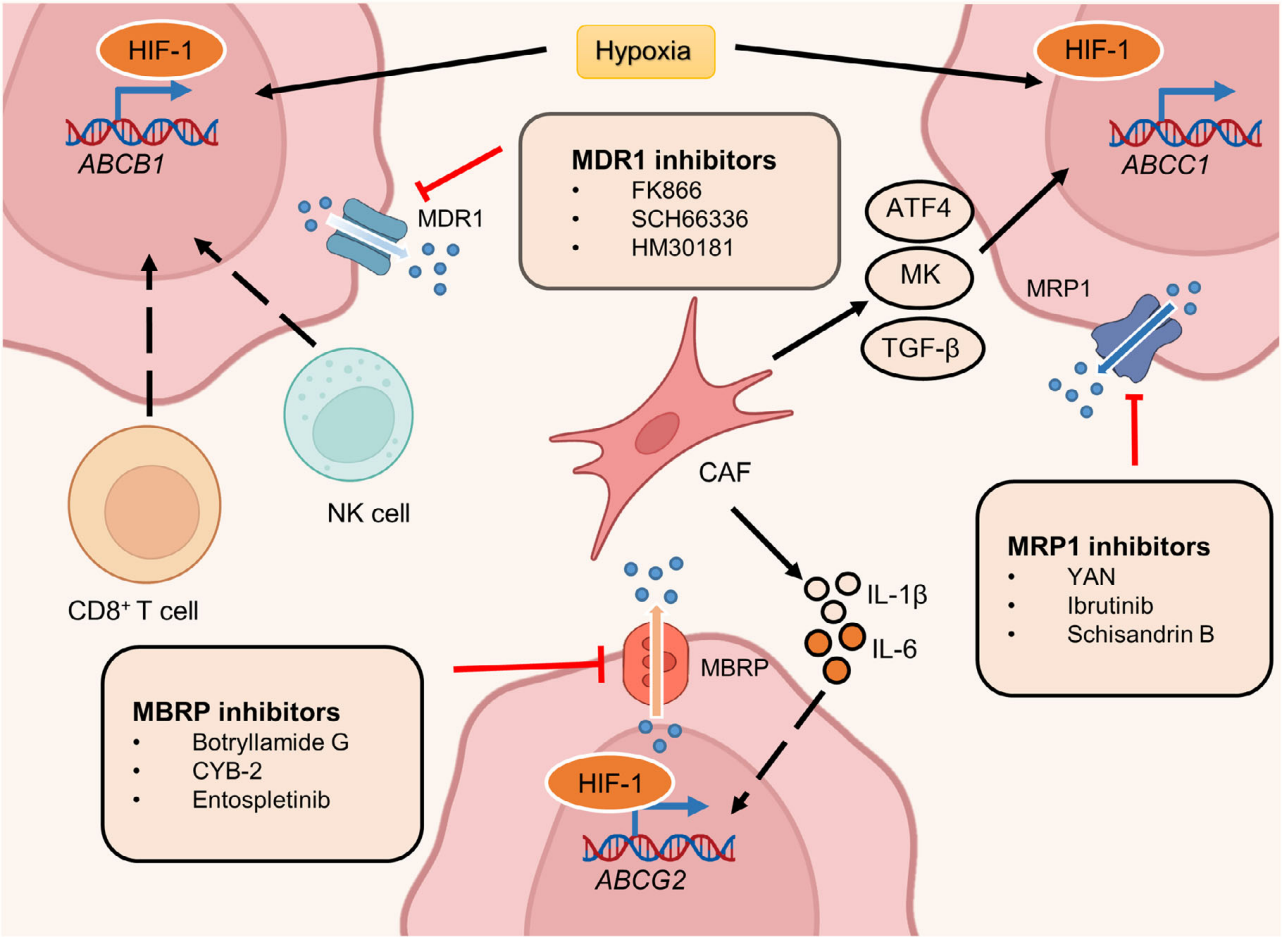
ATP‐binding cassette (ABC) transporter inhibitors and factors that regulate its expression. The transcription factor HIF‐1 is significantly upregulated in the hypoxic niche and subsequently promotes the transcription of *ABCB1, ABCC1*, and *ABCG2*. CAFs, one of the most plentiful stromal components in the TME, induce ABC gene overexpression by secreting growth factors and interleukins. CAF‐derived growth factors, including ATF4, MK, and TGF‐β, enhanced MRP1 expression. Meanwhile, IL‐1β and IL‐6 derived from CAFs transcriptionally activate BCRP expression. Immune cells within the TME, such as NK cells and CD8^+^ T cells, also contribute to *ABCB1* activation, but the underlying mechanism is unknown. The activity of MDR1 could be blocked by many targeting agents, such as FK866, SCH66336, and HM30181. Several inhibitors, such as botryllamide G, CYB‐2, and entospletinib, have been developed to decrease MBRP function. YAN, ibrutinib, Schisandrin B, and other drugs also inhibit MRP1 activation.

The human placenta is generally regarded as the functional barrier between fetal blood circulation and the mother, which protects the fetus from heterologous substances such as therapeutic agents, drugs of abuse, and other xenobiotics circulating in the mother's metabolic system.^254^, ^255^ However, this concept was reconsidered after the thalidomide disaster.^256^ Subsequent research has shown that most xenobiotics and metabolites can cross the placenta and even be transported by the placenta.^257^, ^258^, ^259^ Furthermore, genes involved in the delivery of drugs and metabolites have been identified and termed ABC transporter genes.^260^ In the placenta and the fetal blood–brain barrier (BBB), these transporters can function as efflux pumps of xenobiotics from the maternal circulation.^261^, ^262^ To date, 48 human membrane transporters involved in distinct biological processes have been identified and classified into seven subfamilies. These include the ABC subfamilies A‐G, which regulate the levels of hormones, amino acids, xenobiotics, and other macromolecules by transferring them across cell membranes.^263^, ^264^, ^265^, ^266^ It should be noted that only a small proportion of these transporters have low substrate specificity, with the majority having a much broader spectrum.^267^ This characteristic therefore provides opportunities for cancer cells to develop multidrug resistance. Ample reports suggest that overexpression of ABC transporters enhances the capacity to transport antitumor drugs and is closely associated with drug resistance.^268^, ^269^, ^270^ Among the more than 15 drug resistance‐related ABC transporters identified, multidrug resistance protein 1 (MDR1), also termed P‐glycoprotein or P‐gp, encoded by *ABCB1*, multidrug resistance‐associated protein 1 (MRP1), encoded by *ABCC1*, and breast cancer‐resistant protein (BCRP), encoded by *ABCG2* are thought to be the dominant drug efflux transporters.^271^ Here, we focus on recent studies about these three canonical drug resistance proteins and highlight their dominant functions in resistant cancer cells (Figure 8).

### 5.1 Multidrug resistance protein 1

MDR1 overexpression has been observed in most drug‐resistant cancer cells and is frequently associated with hypoxia.^272^, ^273^ HIF‐1, a hypoxia marker, is significantly associated with MDR1 expression in cancer and normal tissue.^274^, ^275^, ^276^ For example, both physiological and chemical hypoxia can increase the expression of HIF‐1 and MDR1, resulting in doxorubicin resistance in prostate multicellular tumor spheroids.^277^ Conversely, the inhibition of HIF‐1 caused by antisense oligonucleotides (ASOs) significantly decreases MDR1 expression and enhances sensitivity to doxorubicin. The pro‐oxidants H_2_O_2_ and buthionine sulfoximine reduce HIF‐1α and MDR1 expression, indicating the importance of reactive oxygen species (ROS) in drug resistance.^278^, ^279^ In addition to ROS, a high calcium concentration also reduces MDR1 levels by downregulating HIF‐1α expression.^280^, ^281^ Interestingly, in NSCLC hypoxia can reverse doxorubicin resistance by reducing MDR1 expression.^282^ Hence, HIF‐1 may play distinct roles in regulating MDR1 expression under different conditions besides hypoxia.

Ample evidence suggests that MDR1 is also regulated by noncoding RNAs in breast, prostate, lung, pancreatic, and ovarian cancer.^283^, ^284^, ^285^, ^286^, ^287^, ^288^ For instance, CRC patients with high lncRNA CCAL expression have a worse response to adjuvant chemotherapy and shorter overall survival.^289^ The overexpression of CCAL dramatically inhibits activator protein 2α, a suppressor of Wnt signaling, and in turn upregulates MDR1 levels. Additionally, in breast cancer, CCAL can enhance the expression of MDR1 via epigenetic regulation.^290^ Mechanistically, methyltransferase‐like 3 increases the expression of miR‐221‐3p by promoting pri‐miR‐221‐3p m^6^A mRNA methylation, which further triggers the transcription of MDR1 and BCRP.

### 5.2 Multidrug resistance‐associated protein 1

MRP1, the second transporter identified in the ABC transporter family, also plays a crucial role in cancer multidrug resistance.^291^, ^292^, ^293^ MRP1 was found in a small cell lung cancer cell line that showed multidrug resistance.^294^ In 1992, the regulatory gene involved in mediating resistance was first reported in the same cell line.^295^ Since then, the mechanisms by which MRP1 induces drug resistance have been widely revealed. Most of the drug resistance mediated by MRP1 is associated with its aberrant expression.^296^, ^297^, ^298^ Specifically, changes in the extracellular environment, such as oxygen content, can lead to the upregulation of several TFs, which enhances the expression of MRP1.^299^, ^300^, ^301^, ^302^ Hypoxia, one of the most common features of cancer, stimulates the expression of TFs and further augments MRP1 levels in various cancer cells.^303^, ^304^, ^305^ For example, the coexpression of HIF‐1α and MRP1 was observed using immunohistochemical techniques in most of the 50 chordoma specimens, which implied decreased sensitivity to chemotherapy.^306^ However, the mechanism by which HIF‐1α regulates the expression of MRP1 is unclear. A subsequent study of chemoresistant HepG2 cells revealed that ERK/MAPK signaling mediates the activity of HIF‐1α by altering phosphorylation levels.^307^ Active HIF‐1α induces the expression of MDR‐related genes, such as MRP1, and drives chemoresistance. In addition to hypoxia, some CAF‐secreted growth factors can also promote the expression of MRP1.^308^ A recent study indicated that CAF‐derived activating transcription factor 4 (ATF4) positively correlated with malignancy and gemcitabine resistance in PDAC. Further investigation found that CAFs secreted TGF‐β1 to activate the SMAD2/3 pathway, which enhanced the expression of ATF4. Consequently, ATF4 directly bound to the ABCC1 promoter region and upregulated the expression of MRP1.

Midkine (MK), a heparin‐binding growth factor associated with both carcinogenesis and chemoresistance, is another CAF‐derived growth factor that modulates MRP1 levels.^309^ CAFs enhance the expression of lncRNA ANRIL by secreting MK and in turn augment MRP1 expression. Additionally, several metabolites also govern the occurrence of multidrug resistance by upregulating MRP1. In NSCLC, increased glycolysis and lactate production within the TME harmonize the TGF‐β1/Snail and TAZ/AP‐1 pathways to form the Snail/TAZ/AP‐1 complex at the ABCC1 promoter, thus enhancing MRP1 transcription.^310^ The addition of NaHCO_3_ reversed lactate‐induced MRP1 overexpression and overcame etoposide (VP‐16) resistance.

### 5.3 Breast cancer resistant protein

The third identified ABC transporter is BCRP. Similar to the above two transporters, BCRP was detected in numerous types of cancers and contributed to resistance to various antitumor drugs, such as tamoxifen.^311^, ^312^, ^313^ As the most prescribed hormonal agent for the treatment of estrogen receptor α (ERα)‐positive breast cancer, tamoxifen rarely prolongs the overall survival of patients due to drug resistance.^314^ Microarray analysis revealed a higher level of Dicer, an RNase III‐containing enzyme, in tamoxifen‐resistant metastatic breast cancers. Dicer overexpression significantly elevated the level of BCRP and governed resistance to tamoxifen in vivo and in vitro.^311^ Moreover, hypoxia was reported to enhance the stability of HIF, which targeted the promoter of ABCG2 and thus upregulated BCRP expression.^315^ The HIF/BCRP regulatory axis has been discovered in several types of tumors, including breast, ovarian, renal cell, and anaplastic thyroid cancer.^316^, ^317^, ^318^, ^319^, ^320^ In the context of hypoxia and oxidative stress, SP PC populations function to increase the expression of HIF‐2α, which transactivates ABCG2 and promotes cytoprotection.^321^ Recent comprehensive reports have shown crosstalk between N6‐methyladenosine (m^6^A) modification and drug resistance.^322^, ^323^, ^324^ Alteration of the m^6^A modification contributes to the expression of BCRP, which regulates drug efficacy. In addition, CAF‐derived cytokines comprising interleukin 1β (IL‑1β) and interleukin 6 (IL‑6) were also found to promote BCRP expression.^325^, ^326^ These two CAF‐secreted cytokines were detected in breast cancer cells and associated with BCRP‐dependent drug resistance. Glucose can also enhance the expression of BCRP by activating the AKT pathway and subsequently conferring resistance to a small fraction of cancer cells with stem‐like properties, termed side population (SP) cells, within tumors.^327^ However, 3‐BrOP, an inhibitor of glycolysis, could significantly reverse the tumorigenesis ability induced by SP cells, which may provide a potential drug for drug‐resistant SP cells.

It is well known that many types of ABC transporters could be present in a solitary cancer type.^328^ An analysis of the expression of ABC transporters in AML samples showed that coexpression of transporter genes significantly reduced overall survival in patients.^329^ Additionally, quantities of factors enhance the expression of ABC transporters, including gene mutation, epigenetic regulation, and metabolic reprogramming.^330^, ^331^, ^332^, ^333^ Therefore, accurately determining the key ABC transporters involved in drug resistance might provide an approach to benefit clinical outcomes.

## 6 TARGETING ONCOFETAL REPROGRAMMING IN CANCER THERAPY

Understanding the underlying mechanisms of how cancer cells enter the embryonic‐like state is essential to enhance the outcome of patients with drug resistance. In this section, we introduce the inhibitors or potential strategies that target the core factors of embryonic development‐related signaling pathways, transporter proteins, the drivers of dormant cancer cells, and immune cell subpopulations implicated in immunotherapeutic resistance.

### 6.1 Targeting developmental signaling pathways

Dysregulated developmental pathways are commonly detected in cancer cells and proven to mediate drug resistance and recurrence. Given its critical functions, many targeting agents have been developed to aid cancer treatment.

Wnt signaling pathway inhibitors can be grouped into four main categories: ligand or receptor inhibitory agents involved in Wnt signaling; porcupine antagonists that target the processing or secretion of Wnt ligands; agents that maintain the function of the destruction complex by activating caspases or inhibiting tankyrase, thereby enhancing the deregulation of β‐catenin; and blockades of β‐catenin–TCF axis‐dependent transcription^334^, ^335^, ^336^ (Figure 2). To date, each class of agents has achieved good results in preclinical studies, and several drugs have entered clinical testing (Table 1). In the canonical Wnt pathway, inhibition of FZD receptors is linked to β‐catenin deregulation and suppression of Wnt signaling, which indicates a potential strategy for oncotherapy.^337^, ^338^ To achieve this goal, several target agents, such as ipafricept,^339^ OMP‐18R5 (vantictumab),^340^, ^341^, ^342^ OTSA‐101,^343^ F2. A,^344^ and Fz7‐21,^345^ have been utilized to competitively bind to the FZD family, thereby inhibiting Wnt‐dependent oncogenesis. In addition, DKN‐01, a mAb to DKK1 that blocks Wnt signaling by enhancing the internalization of low‐density lipoprotein (LDL)‐related protein 5/6 (LRP5/6) coreceptors, has entered clinical trials.^14^, ^346^, ^347^ The development of porcupine antagonists is another approach to suppress the Wnt pathway by affecting the key factor for the secretion of Wnt ligands.^348^, ^349^ For instance, WNT974 (LGK974) has been proven to abolish Wnt secretion and exert effective antitumor activity in epithelial ovarian cancer.^350^, ^351^ WNT974 monotherapy for patients with cervical squamous cell carcinoma, pancreatic cancer, and triple‐negative breast cancer (TNBC) has entered a phase I clinical trial (NCT01351103).^352^ In preclinical or clinical trials, β‐catenin is one of the most common targets of inhibitors due to its pivotal role in Wnt signaling. CWP232291, a first‐in‐class peptidomimetic drug, decreases β‐catenin by activating caspases and shows anticancer activity against relapsed or refractory myeloma.^353^ Moreover, several compounds, such as LF3 and KYA1797K/KY1220, also effectively decrease the activation of Wnt pathway by targeting the β‐catenin/TCF complex.^354^, ^355^, ^356^ In preclinical studies, both LF3 and KYA1797K/KY1220 effectively suppress the growth of colon cancer by disrupting the critical interaction between β‐catenin and TCF4.^357^, ^358^

**TABLE 1:** Small molecules targeting Wnt signaling and their clinical trials.

Compound	Tumor type	Phase (Clinicaltrials.gov identifier)	Efficacy outcomes	Status	References
PRI‐724 with gemcitabine	Advanced metastatic pancreatic cancer	Phase Ib (NCT01764477)	8 patients had SD (40.0%)	Clinical studies ongoing	^359^
DKN‐01	Advanced‐stage DKK1‐positive esophageal cancer or gastroesophageal junction tumors	Phase Ib study (NCT02013154)	Encouraging early efficacy signals (no further details reported)	Clinical studies ongoing	^360^
Ipafricept	Advanced‐stage solid tumors	Phase I (NCT01608867)	3 patients had SD for more than 6 months (1 with germ cell cancer and 2 with desmoid tumor)	Clinical studies ongoing	^361^
Vantictumab	Advanced solid tumors	Phase I	SD in 3 patients with NET	Clinical studies ongoing	^362^
Vantictumab	Stage IV pancreatic ductal adenocarcinoma	Phase I (NCT02005315)	53% evaluable patients (8 out of 15) had a PR and 27% had SD (4); Median PFS: 7.2 months	Clinical studies ongoing	^363^
Vantictumab	Metastatic HER2‐negative breast cancer	Phase Ib (NCT01973309)	33% evaluable patients (7 of 21) had a PR and 29% (6) had SD	Updated results pending	^364^
Cirmtuzumab	Chronic lymphocytic leukemia	Phase I (NCT02222688)	17 of 22 evaluable patients had SD	Clinical studies ongoing	^365^
OTSA101	Synovial sarcoma	Phase I (NCT01469975)	77% evaluable patients (3 of 8) had SD	Recruiting	^343^
CWP232291 alone or with lenalidomide and dexamethasone	Myeloma (relapsed or refractory)	Phase Ia/b (NCT02426723)	27% patients (3 of 11) had SD	Clinical studies ongoing	^366^
CWP232291 (CWP291)	Relapsed and/or refractory AML or myelodysplastic syndrome	Phase I (NCT01398462)	1 CR in a patient with AML	Clinical studies ongoing	^353^

Abbreviations: AML, acute myeloid leukemia; CR, complete response; DKK1, Dickkopf‐related protein 1; Fzd, Frizzled; *n*, number of patients; NET, neuroendocrine tumor; PFS, progression‐free survival; PR, partial response; SD, stable disease.

The development of Notch signaling inhibitors necessitates a thorough understanding of its diverse and complex role in various cancers^367^, ^368^, ^369^ (Figure 3). Given the importance of Notch signaling pathways in cancer progression, such as angiogenesis and stemness maintenance, several classes of agents have been developed that target the Notch pathway in distinct ways^370^, ^371^ (Table 2). The γ‐secretase inhibitors (GSIs) are the first and largest class of small molecule antagonists that block the second proteolytic cleavage of Notch receptors and thus inhibit the activation of Notch downstream.^372^ GSIs exert strong antitumor activities in many preclinical trials. For instance, the combination of MRK‐003 and trastuzumab fully eliminated HER2‐positive breast cancer cells in a mouse model.^373^ Moreover, in an NSCLC model, another GSI, BMS‐906024 also displayed synergistic therapy potential when combined with cisplatin, crizotinib, PTX, and docetaxel.^374^, ^375^ Notch ligands are also targets for antitumor drug development. A phase I first‐in‐human study of enoticumab, a human anti‐DLL4 IgG1 mAb, showed potential therapeutic effect in patients with advanced solid tumors.^376^ In addition to enoticumab, many other humanized antibodies that target DLL‐3, such as rovalpituzumab tesirine, have also been tested in clinical trials and have shown signs of efficacy in patients with recurrent small cell lung cancer.^377^ Monoclonal antibodies targeting Notch receptors (such as brontictuzumab and tarextumab) have been well demonstrated to show moderate antitumor activities in different phases of studies.^378^, ^379^, ^380^ CB‐103 is a novel small molecule inhibitor that targets Notch signaling by disrupting the interaction of the Notch transcription complex.^381^ Preclinical studies showed that CB‐103 significantly inhibited the growth of tumors in a GSI‐resistant TNBC model. Moreover, another inhibitor targeting the Notch coactivator protein, IMR‐1, inhibits the growth of Notch‐dependent cell lines and patient‐derived tumor xenografts.^380^

**TABLE 2:** Small molecules targeting Notch signaling and their clinical trials.

Compound	Tumor type	Phase (Clinicaltrials.gov identifier)	Efficacy outcomes	Status	References
MK‐0752	T‐ALL and AML	Phase I (NCT00100152)	1 patient with T‐ALL had a 45% reduction in a mediastinal mass	Discontinued	^382^
MK‐0752 with gemcitabine	Unresectable PDAC	Phase I (NCT01098344)	47% patients had SD (9 of 19); PRs: 5% patients (1 of 19)	Discontinued	^383^
MK‐0752 followed by docetaxel	Advanced‐stage breast cancer	Phase I/II (NCT00645333)	Not reported	Discontinued	^384^
MK‐0752 with ridaforolimus	Advanced‐stage solid tumors	Phase I (NCT01295632)	11% patients (2 of 18) with HNSCC had responses; 1 patient had SD lasting ≥6 months	Discontinued	^385^
PF‐03084014	Advanced‐stage solid tumors	Phase I (NCT00878189)	CR: 2% evaluable patients (1 of 46; thyroid cancer); PRs: 11% patients (5 of 46; all desmoid tumors); 30% patients had SD (14 of 46)	Clinical studies ongoing	^386^
PF‐03084014	Advanced‐stage TNBC	Phase II (NCT02299635)	Not reported	Clinical studies ongoing	^387^
PF‐03084014	T‐ALL or T‐LBL	Phase I (NCT00878189)	CR: 12.5% patients (1 of 8)	Clinical studies ongoing	^388^
PF‐03084014	Desmoid tumors	Phase II (NCT01981551)	PR: 29% patients (5 of 16); 29% patients had SD (5 of 16)	Clinical studies ongoing	^389^
PF‐03084014 with gemcitabine and nab‐paclitaxel	Metastatic PDAC	Phase Ib/II (NCT02109445)	ORR: 0%	Clinical studies ongoing	
RO4929097	Metastatic CRC	Phase II (NCT01116687)	18.2% evaluable patients had SD (6 of 33); Median OS: 6.0 months; Median PFS: 1.8 months	Discontinued	^390^
RO4929097 with gemcitabine	Advanced‐stage solid tumors	Phase I	PR: 5.6% patient with nasopharyngeal carcinoma; 3 patients had SD	Discontinued	^391^
RO4929097 with temsirolimus	Advanced‐stage solid tumors	Phase Ib (NCT01198184)	73% patients had SD	Discontinued	^392^
BMS‐906024	T‐ALL	Phase I	(32%) patients (8) had ≥50% reduction in bone marrow blasts	Clinical studies ongoing	^393^
Brontictuzumab	Hematological malignancies	Phase I	PR in 4.3% patient with TMF (1); 8.7% patients had SD in (2)	No phase II or III trials ongoing; discontinued in hematological malignancies	^394^
Tarextumab or placebo with gemcitabine and nab‐paclitaxel	Metastatic PDAC	Phase Ib/II (NCT01647828)	Placebo vs. tarextumab arms: Median OS: 7.9 months vs. 6.4 months Median PFS: 5.5 months vs. 3.7 months; ORR: 31.8 vs. 20.2%	Discontinued	^395^
Tarextumab or placebo with etoposide and cisplatin	Extensive‐stage SCLC	Phase Ib/II (NCT01859741)	ORR in 84% patients of phase Ib part; placebo vs. tarextumab arms in phase II part: Median PFS: 10.3 months vs. 9.3 months; ORR: 70.8 vs. 68.5%;	Discontinued	^396^
Demcizumab or placebo with carboplatin and pemetrexed	Metastatic nonsquamous NSCLC	Phase I (NCT01189968)	3% patients had CR patients (1 of 40); 48% patients had PR (19 of 40); 38% patients had SD (15 of 40)	Progressed to the randomized phase II study (NCT02259582)	^397^
Demcizumab with gemcitabine, with or without nab‐paclitaxel	Advanced‐stage PDAC	Phase Ib (NCT01189929)	50% evaluable patients had PR (14 of 28); 39.3% patients had SD (11 of 28); Median OS: 10.1 months; Median PFS: 9.0 months	Progressed to the randomized phase II study (NCT02289898)	^398^
Rovalpituzumab tesirine	SCLC	Phase I (NCT01901653)	17% patients had PR or CR (11 of 65 patients); 54% patients had SD (35 of 65); Median OS: 4.6 months; Median PFS: 3.1 months	Progressed to the phase II study (NCT02674568)	^377^

Abbreviations: AML, acute myeloid leukemia; CR, complete response; CRC, colorectal cancer; DoR, duration of response; HNSCC, head and neck squamous cell carcinoma; *n*, number of patients; NSCLC, non‐small cell lung cancer; ORR, objective response rate; OS, overall survival; PDAC, pancreatic adenocarcinoma; PFS, progression‐free survival; PR, partial response; SCLC, small‐cell lung cancer; SD, stable disease; T‐ALL, T‐cell acute lymphocytic leukemia; T‐LBL, T‐cell lymphoblastic lymphoma; TMF, transformed mycosis fungoides; TNBC, triple‐negative breast cancer.

Among the components involved in the Hedgehog pathway, its ligands, Smo and Gli transcription factors, are the most common targets in Hedgehog pathway‐directed oncotherapy^399^, ^400^, ^401^ (Figure 4). Agents that target Smo and Gli are currently in clinical trials^402^ (Table 3). Vismodegib and sonidegib are two United States Food and Drug Administration‐approved antagonists of SMO for treating patients with metastatic BCC and/or recurrent locally advanced BCC.^403^ These two Hedgehog pathway inhibitors yielded overall objective response rates (ORRs) from 44 to 68% in different stages of clinical trials.^403^, ^404^, ^405^, ^406^, ^407^ Glasdegib, combined with low‐dose cytarabine to treat patients with newly diagnosed AML aged over 75 years old and ineligible for intensive induction chemotherapy, was an important advance in the development of SMO inhibitors in 2018.^408^ Meanwhile, retarding transcriptional activity of Hedgehog signaling is an attractive therapeutic option, and agents targeting Gli‐mediated transcription, such as GANT58 and GANT61a, are utilized to overcome cancer resistance to SMO inhibitors.^409^, ^410^ GANT61 exhibited significant antitumor activity in several preclinical models, including breast, ovarian, pancreatic, lung, and liver cancer.^411^, ^412^, ^413^, ^414^ In addition, GANT58 decreased the growth of prostate cancer cells by suppressing the expression of PTCH1 and Gli1 in vivo.^415^

**TABLE 3:** Small molecules targeting Hedgehog signaling and their clinical trials.

Compound	Tumor type	Phase (Clinicaltrials.gov identifier)	Efficacy outcomes	Status	References
Vismodegib vs. placebo	Recurrent epithelial ovarian, primary peritoneal cancer or fallopian tube in second or third CR	Phase II (NCT00739661)	Median PFS: 7.5 months vs. 5.8 months with placebo	Magnitude of sought increase in PFS not achieved	^416^
Vismodegib or placebo in combination with FOLFOX or FOLFIRI plus bevacizumab	mCRC	Phase II	PFS: HR 1.25, 90% CI 0.89−1.76, *p* = 0.28; 1‐year OS: 81.4 vs. 80.1%; ORR: 51% in vismodegib arm vs. 46% in placebo arm	Development for CRC terminated	^417^
Vismodegib with gemcitabine	Advanced‐stage PDAC (25 with elevated SHH on pretreatment biopsy)	Phase II (NCT01195415)	Fibrosis decreased in 45.4% of 22 patients and Ki67 levels decreased in 52.9% of 17 patients; *Gli1* and *PTCH1* expression decreased in 95.6 and 82.6%, respectively, of 23 evaluable patients; Median OS: 5.3 months; DCR: 65.2%; Median PFS: 2.8 months; ORR: 21.7%	Development for PDAC terminated	^418^
Vismodegib or placebo with gemcitabine	Advanced‐stage PDAC	Phase I/II (NCT01064622)	Median OS: 6.9 months vs. 6.1 months (HR 1.04, 95% CI 0.69−1.58); PR rate: 8 vs. 11%; SD rate: 51 vs. 38%; Median PFS: 4.0 months vs. 2.5 months (HR 0.81, 95% CI 0.54−1.21); CR rate: 0% in vismodegib arm vs. 2% in placebo arm	Development for PDAC terminated	^419^
Vismodegib preoperatively and/or postoperatively	Recurrent resectable glioblastoma	Phase II (NCT00980343)	Median PFS and OS were 1.8 months and 8.3 months, respectively	Development for glioblastoma terminated	^420^
Vismodegib or placebo with FOLFOX	Advanced stage gastric or gastroesophageal junction adenocarcinoma	Phase II (NCT00982592)	Median PFS: 7.3 months vs. 8.0 months in the placebo group; ORR: 35% in both arms; Median OS: 11.5 months vs. 14.9 months with placebo	Development terminated for these diseases	^421^
Taladegib	Advanced‐stage cancer	Phase I (NCT01226485)	ORR: 26.2%; SD rate: 28.6%;	Clinical studies ongoing	^407^
Saridegib	Advanced‐stage solid tumors	Phase I	6 PRs observed in 22 patients with HH inhibitor‐naïve BCC (27%)	Clinical studies ongoing	^422^
Saridegib (with cetuximab)	Recurrent metastatic head and neck squamous cell carcinoma	Phase I (NCT01255800)	Median PFS: 77 days; 12.5% evaluable patients had PR (1 of 8) and 50% had SD (4 of 8)	Clinical studies ongoing	^423^
Itraconazole	Biochemically relapsed prostate cancer	Phase II (NCT01787331)	47% evaluable patients (9 of 19) had PSA declines by week 12	Development for prostate cancer terminated	^424^
Itraconazole and pemetrexed	Metastatic nonsquamous non‐small cell lung cancer	Phase II	15 patients received pemetrexed and itraconazole and 8 received pemetrexed alone); OS: 32 months vs. 8 months; Median PFS: 5.5 months vs. 2.8 months	Discontinued	^425^
Itraconazole with arsenic trioxide	Refractory metastatic BCC	Phase I	SD in 3 patients	Study ongoing, further results pending	^426^

Abbreviations: ALT, alanine transaminase; AML, acute myeloid leukemia; AST, aspartate transaminase; BCC, basal cell carcinoma; CR, complete response; CRC, colorectal cancer; DCR, disease control rate; DoR, duration of response; FOLFIRI, folinic acid, 5‐fluorouracil and irinotecan; FOLFOX, folinic acid, 5‐fluorouracil and oxaliplatin; HH, Hedgehog; HR, hazard ratio; mCRC, metastatic colorectal cancer; ORR, objective response rate; OS, overall survival; PDAC, pancreatic ductal adenocarcinoma; PFS, progression‐free survival; PR, partial response; PSA, prostate‐specific antigen; SD, stable disease; SHH, Sonic hedgehog.

LATS1/2 and MST1/2 activators are currently the most used agents in Hippo‐targeted therapeutics^427^, ^428^ (Figure 5). Due to the distinctive characteristics of the Hippo pathway, in which loss‐of‐function mutations of LATS1/2 and MST1/2 are always linked to oncogenesis, the development of targeted agents presents more challenges than other antagonists.^429^, ^430^ Indeed, to date, only a few agents specifically targeting Hippo signaling have been brought into clinical testing. For example, pevonedistat, a first‐in‐class NEDD8‐activating enzyme inhibitor, blocks CRL4DCAF1‐mediated degradation of LATS1/2 and thereby attenuates YAP/TAZ activity.^431^, ^432^ Pevonedistat has been tested in combination with azacitidine in a phase I trial involving patients with AML or MDS, which resulted in 83% attenuation of ORRs in patients who received more than 6 cycles of treatment.^156^

A variety of receptor kinase inhibitors, monoclonal antibodies, ligand traps, and ASOs have been developed to block TGF‐β signaling, and most agents have been or are being tested in clinical trials to provide more effective treatments (Figure 6). The small molecule inhibitors of TGF‑β receptor kinase were mainly designed to bind to the ATP‐binding domain of TGF‐β R kinase, therefore inhibiting ATP kinase activity and abolishing the downstream signaling cascade.^102^ For example, vactosertib (MedPacto), an oral inhibitor that targets TGF‐βRI/ALK‐5 (IC50 = 12.9 nM), acts pleiotropically on diverse cancer types, including CRC, gastric cancer, and NSCLC, through intrinsic and extrinsic mechanisms.^433^, ^434^ Galunisertib is another kinase inhibitor that has shown antitumor effects in lung and breast cancer cell lines, and its safety was proven in phase I studies.^435^, ^436^ Moreover, many other agents targeting TGF‐βRI, such as LY3200882, LY573636, and A83‐01, have also been reported to display certain antitumor activity in various tumors by weakening the activity of TGF‐β signaling.^437^, ^438^, ^439^ Another potential method for reversing the tumorigenic and immune suppressive responses induced by the TGF‐β pathway is the administration of antibodies that can obstruct the binding of TGF‐β ligand to its receptor. Indeed, the highly selective inhibitor of the TGF‐β1 isoform SRK181‐mIgG1 was shown to overcome primary resistance to checkpoint inhibitor therapy, such as anti‐PD‐(L) 1 Abs.^440^ Meanwhile, in a phase I trial of 28 patients with malignant melanoma, Fresolimumab, a type of human mAb that neutralizes TGF‐β1 and TGF‐β2, showed a partial response in 25% of patients.^441^ The development of TGF‑β ligand traps is an approach to handle the exogenous‐dependent hyperactivation of TGF‑β signaling.^442^ In this context, cancer patients might gain more precise treatment. Currently, there is an increasing number of ways to inhibit the TGF‑β pathway, such as ASOs and vaccine‑based approaches to modulate TGF‑β signaling, which again demonstrates their irreplaceable role in cancer.^443^, ^444^

Most FGF/FGFR signaling inhibitors fall into three groups: small‐molecule FGFR TKIs, anti‐FGFR antibodies, and FGF ligand traps^445^ (Figure 7). The most widely used therapeutic approach are FGFR TKIs, which include pan‐FGFR inhibitors (such as FIIN‐2, JNJ‐42756493, LY2874455, and ponatinib) and FGFR‐specific TKIs (such as the selective FGFR4 inhibitor BLU9931).^446^ Furthermore, according to the interaction pattern between the inhibitor and the kinase domain, FGFR TKIs can be classified into irreversible or reversible inhibitors. Irreversible inhibitors (such as erdafitinib and pemigatinib) are thought to have a better binding affinity and selectivity, but the early phases of clinical trials showed limited efficacy or demonstrated minimal clinical benefit.^447^, ^448^ Other molecules that have been developed as investigational agents targeting FGF/FGFR signaling include the FGF traps FP‐1039 (HGS1036), msFGFR2c, sFGFR3 sm27, and NSC12^449^, ^450^, ^451^; the anti‐FGF2 mAbs 3F12E7^452^ and H3L3^453^; the anti‐FGFR2 mAb hFR2‐14^454^; the anti‐FGFR4 mAb U3‐1784^455^; and the anti‐FGFR1 antibody–drug conjugate (ADC) LY3076226.^456^ The benefits of FGFR inhibitors have been proven in clinical trials in subsets of patients, including those with lung, breast, and gastric cancer. However, the low response rates among patients with FGFR alterations and the existence of responders without detectable FGFR alterations still hamper treatment outcomes.

### 6.2 Eliminating dormant cancer cells

Previous comprehensive reviews have summarized the therapeutic strategies to change the TME and improve therapy outcomes.^457^, ^458^, ^459^, ^460^, ^461^ Here, we focus on advances in the field of dormant cancer cells‐related treatment by altering the TME. Forcing dormant cancer cells to enter distinct oxygen conditions, such as the switch between chronic hypoxia and acute hypoxia, has been proposed to improve the efficacy of antitumor drugs.^462^, ^463^ The combined treatment showed that the vitamin B3 analog nicotinamide, which prevents temporary fluctuations in tumor blood flow, increases the oxygen level and shrinks the extent of tumor metastasis.^464^ In addition, mild temperature hyperthermia, another approach to elevate tumor blood flow, was reported to relieve acute hypoxia and enhance drug susceptibility.^465^ Altering hypoxia at various degrees of irradiation increased radiosensitivity, particularly to X‐rays.^466^ For example, nicotinamide or mild temperature hyperthermia enhances the radiosensitivity of total and dormant cancer cells when given X‐rays in combination with high‐dose‐rate irradiation.^462^, ^467^, ^468^ Therefore, irradiation combined with nicotinamide or mild temperature hyperthermia could significantly improve the patient's prognosis. Moreover, bevacizumab, a drug targeting vascular endothelial growth factor (VEGF), impairs oxygen supply and shows antitumoral effects in various drug‐tolerant persister cells.^469^, ^470^, ^471^ Given the roles of VEGF in the formation of blood vessels, bevacizumab could dramatically augment the hypoxic niche by influencing oxygen transport and exerting greater antineoplastic activity when combined with chemotherapy.^472^, ^473^ However, in patients with refractory breast cancer, the addition of bevacizumab to PTX treatment seems to be ineffective under hypoxic conditions and may induce hypoxia and increase cytokine secretion related to cancer progression.^474^ These findings suggest that targeting hypoxia might be a promising way to eradicate tumor cells and dormant cancer cells, although many unknown mechanisms need further study.

A substantial body of evidence suggests the crucial roles of TAMs in inducing chronic inflammation and contributing to cancer development.^475^, ^476^, ^477^ The inflammatory microenvironment is now recognized as one of the hallmarks of cancer^478^ and tightly correlated with dormant malignant cells due to its multifaceted functions in retaining tumor dormancy and reawakening dormant tumor cells.^479^, ^480^, ^481^ Hence, macrophage‐targeting therapeutic strategies might be a promising way to change the dormant state of cancer cells and thus inhibit tumor relapse. A number of potential strategies targeting macrophages have been reported.^482^, ^483^, ^484^ In general, TAM‐focused therapeutic approaches are classified into either restraining the interplay by inhibiting the localization of these cells at tumor sites or reactivating their antineoplastic activities. As previously discussed, chemokines (such as CCL5) are responsible for the recruitment of macrophages to tumors.^485^ In research on residual breast cancer cells, Her2 downregulation‐driven CCL5 has been reported to promote breast cancer recurrence via macrophage recruitment.^486^ CCL5, a product of cancer cells and macrophages, is usually linked to a worse outcome in diverse types of breast cancer.^487^, ^488^ Blocking CCL5 with maraviroc, the cognate receptor for CCL5, is associated with biological and clinical responses in advanced stage CRC.^489^ Thus, drugs targeting the CCL5–CCR5 axis are worthy of further study, considering that the functions of this signaling pathway are well established in the pathogenesis of breast cancer, GBM, and gastric cancer.^490^, ^491^, ^492^ CSF‐1R, the key modulator of monocyte‐macrophage lineage growth and differentiation, is abundantly expressed in numerous tumor types.^493^, ^494^, ^495^ Given the direct interference with TAMs, CSF­1R has become a promising therapeutic target, and several related inhibitors (such as small molecules and antibody antagonists) have been tested in different preclinical models.^496^, ^497^, ^498^ For instance, in glioma macrophage populations after radiotherapy, a CSF‐1R inhibitor combined with radiotherapy enhanced the outcome in preclinical models, accompanied by decreased recruitment of monocyte‐derived macrophages in GBM.^499^ Moreover, pexidartinib, a drug used for recurrent GBM, can reduce the number of circulating CD14dim/CD16+ monocytes. Although the primary 6‐month progression‐free survival (8.6%) was not satisfactory in the 37 patients after treatment, rational combination therapy approaches may significantly augment the outcome.^500^ In general, macrophage‐targeting therapeutic strategies have the potential to complement and synergize with both chemotherapy and immunotherapy.

### 6.3 Regulation of tumor immune microenvironment

Reciprocal communication between cancer cells and different immune cell subpopulations enables tumor cells to escape immune responses and develop resistance to ICBs.^501^, ^502^, ^503^ Hence, the heterogeneity of the immune microenvironment is tightly linked to the outcomes of ICB in immunotherapy. Combined treatment was used to address this issue, considerably enhancing the treatment effect.

In a phase III GBM trial, regulatory Tregs were shown to play crucial role in resistance to ICBs.^504^ Therefore, targeting glucocorticoid‐induced TNFR‐related receptor (GITR) in Treg cells using an agonistic antibody (αGITR) significantly reversed the suppression of antitumor immune response. The functionality of intratumoral macrophages can reflect the response to ICBs in melanoma. Mechanistically, the activation of ER promotes melanoma growth in murine models by skewing macrophage polarization, which leads to ICB resistance.^505^ Targeting ER by using fulvestrant, a selective ER inhibitor greatly decreased tumor growth and promoted the antitumor efficacy of ICBs. Nanobased combinational treatment is also a potent way to target immunotherapeutic resistant cancer cells by reprogramming the immune environment. To overcome tumor immunological tolerance against ICBs, a versatile nanomodulator was designed that could point‐to‐point counteract immune suppressors and promote the infiltration of tumor T cells.^506^ Small interfering RNAs targeting indoleamine 2, 3‐dioxygenase‐1 and gemcitabine delivered by biocompatible nanocages account for targeting Tregs and MDSCs. Meanwhile, O_2_‐producible mineralization tattooed on the surface of the nanocarriers accounts for suppressing the immune inhibition of M2 macrophages. A multifunctional nanomodulator was used to reverse the immunosuppressive state and overcome tumor immunological tolerance after decorating the therapeutic ICB antibodies on the mineralized shell. In many situations, targeting the tumor immune environment may be feasible to decrease resistance to ICBs.

### 6.4 Inhibition of ABC transporters

Targeting ABC transporters has been considered a promising approach to suppressing or eliminating drug resistance in oncotherapy since discovering the vital role of ABC proteins in tumor drug resistance.^507^, ^508^ As early as 1981, Tsuruo's group found that verapamil could weaken drug resistance in leukemia cells.^509^ However, high dosages of verapamil trigger cardiovascular toxicity, limiting its further applications.^510^ The second generation of P‐gp inhibitors, such as dexverapamil,^511^ valspodar (PSC 833),^512^ and biricodar citrate (VX‐710),^513^ failed to cause drug–drug interactions with other antineoplastic agents, as did the first generation.^514^, ^515^, ^516^ Continuing failures with antidrug resistance agents have driven the development of third‐generation inhibitors. These novel drugs are not only more than 200‐fold more potent at reversing drug resistance than before but also block the interaction with other chemotherapeutic drugs.^517^, ^518^ For example, tariquidar (XY9576), a drug currently in clinical trials, binds to P‐gp with high affinity and inhibits its ATPase activity at very low concentrations.^519^ Clinical trials have demonstrated tariquidar's potential as a candidate, even if additional studies indicated that it might work in vivo either as a substrate or an inhibitor of P‐gp depending on the dosage.^520^

With the development of nanotechnology approaches, novel strategies for targeting ABC transporters have been established, paving the way for more effective treatment of drug‐resistant cancer.^521^, ^522^, ^523^ The diameter of nanoparticles (NPs) varies from one to several hundred nm. NPs can load a range of antitumor molecules, such as antineoplastic agents, P‐gp inhibitors, and RNAi fragments.^524^, ^525^ Mesoporous silica material (MSNPS)‐based drug delivery is one example, as it has a high surface area, large pore volume, biocompatibility, and tunable pore size. MSNPS loaded with siRNA and anticancer drugs showed significant antitumor activity by inhibiting P‐gp expression and increasing intracellular drug concentrations.^526^

## 7 CONCLUSIONS

During the development of mammals, fetal stem cells can differentiate into diverse types of cells to ensure normal body function.^527^, ^528^ These observations imply that cells can switch from one lineage to another, greatly expanding our understanding of cell identity and fate determination. Recently, this hallmark of cancer cells has been comprehensively reviewed by Hanahan and termed unlocking phenotypic plasticity.^529^ In his opinion, cellular plasticity could be selectively regulated by three mechanisms: dedifferentiation, blocked differentiation, and transdifferentiation, which are tightly linked to carcinogenesis. Indeed, to encounter diverse pressures from diverse niches, cancer cells can alter their state to gain stronger adaptability.^530^, ^531^ Embryonic development is a special phase in the progression from a fertilized egg to a mature individual.^532^ On the one hand, embryos need to activate developmental signaling pathways to ensure their rapid proliferation and accurate differentiation.^84^, ^533^, ^534^, ^535^ On the other hand, deficiency in embryonic detoxification systems requires more effective efflux of toxic substances, which is mainly performed by ABC transporters.^536^, ^537^ In some harsh circumstances, the embryo will even stop developing until favorable conditions are restored.^538^, ^539^ Meanwhile, fetuses can regulate the immune microenvironment to evade attacks from the maternal immune system.^540^, ^541^, ^542^, ^543^, ^544^

Cancer therapy is still a major challenge because each patient with cancer has their underlying mechanisms of tumorigenesis, metastasis as well as resistance, and there is a defining set of characteristics that dictate tumor deterioration, which may eventually result in death.^545^, ^546^, ^547^ With the continuous advancement in cancer research, many new drugs and treatment approaches have emerged in cancer therapy, including multiple drug delivery‐based nanocarriers, CAR‐T or ICB immunotherapy, CRISPR‐based gene therapy, and so on. Though these approaches have shown significant potential in clinical studies, not all cancer patients can benefit from the advances. For example, CAR‐T therapy has demonstrated promising results in the treatment of hematological malignancies such as ALL and lymphoma, but its efficacy is significantly reduced in solid tumors.^548^ Therefore, there is a growing awareness of the critical role of tumor heterogeneity in cancer therapy and the importance of precision medicine. Precision medicine in cancer care offers numerous advantages, including personalized treatment tailored to an individual's genetic and biological characteristics, minimizing side effects, increasing treatment success rates, avoiding trial‐and‐error approaches, early risk prediction and prevention measures, real‐time treatment monitoring, and contributing to future cancer research through data collection. However, the development of precision medicine must be built upon the foundation of preclinical research and clinical translation. Future studies need to focus on the mechanistic links between cellular stress sensors and embryo‐like transitions in different molecular contexts and tumor types, which enabling better understanding of the complexity and heterogeneity of tumors and facilitates the development and application of novel treatment approaches.

It is well understood that abnormal genetic or nongenetic alterations in normal cells can drive cancer onset and progression. However, the described changes can be viewed as a process of empowering tumor cells, enabling them to possess a range of malignant functions, including resistance to immune clearance, sustenance of their own growth, and the capability to metastasize within the body. To date, researchers have made significant strides in understanding that cancer cells exhibit the remarkable capacity to mimic various specialized cell types. They can, for example, emulate neutrophils by entering the circulatory system and infiltrating different organs, engage in asymmetric differentiation akin to stem cells, and even establish communication with the nervous system.^540^, ^541^, ^542^ For these reasons, many scientists now believe that the genesis of cancer cells is, in fact, a form of atavism.^543^ Meanwhile, the concept of oncofetal reprogramming has been proposed due to the striking resemblance between cancer cells and the high pluripotency potential exhibited by cells during early embryonic development in various higher animals.^544^ Here, we have discussed representative embryonic development hallmarks contributing to tumor development and progression via genetic, epigenetic, or TME alterations. In addition, investigating the intricate mechanisms by which tumor cells respond to embryonic developmental genes and pathways can lead to new therapeutic approaches for cancer treatment in patients, increasing the possibility of a cure. Although methods for solving this problem appear challenging, we systematically revisit cancer development from an embryonic development perspective, which provides new insight into this intricate issue. Despite the disadvantages, it does seem feasible, at least conceptually, to draw a roadmap for confronting the problem of cancer therapy.

## AUTHOR CONTRIBUTION

J. C., Z. Z., and L. Z. searched for original articles and wrote the manuscript. M. L., L. L., and B. L. prepared the figures. E. C. N. revised the manuscript. Z. S., W. H., and C. H. conceived and critically discussed the clinical unmet problems, underlying mechanisms and wrote the manuscript. All authors have read and approved the final version of the manuscript.

## CONFLICT OF INTERESTS STATEMENT

Author Canhua Huang is an Editorial board member of Medcomm. Author Canhua Huang was not involved in the journal's review of or decisions related to this manuscript. The other authors declared no conflict of interest.

## ETHICS STATEMENT

Not applicable.

## Data Availability

Not applicable.
